# A Bottleneck Analysis of Iron and Folic Acid Supplementation Program in Pakistan

**DOI:** 10.1111/mcn.13797

**Published:** 2025-02-11

**Authors:** Anamta Ghur, Soha Randhawa, Namira Shahid, Waffa Ali, Banafsha Ali, Saroosh Rashid, Ambreen Khan, Aman Akbar Babri, Khawaja Masood, Saba Shuja, Maliha Fatima, Noshad Ali, M. Imran Khan, Ali Turab

**Affiliations:** ^1^ Global Health deaprtment PHC Global Karachi Pakistan; ^2^ Health department Nutrition Wing—Ministry of National Health, Services, Regulations, and Coordination (MoNHSRC) Islamabad Pakistan; ^3^ Health sector UNICEF Islamabad Pakistan

**Keywords:** anemia, iron deficiency anemia, iron folic acid service delivery, iron supplements, NVivo, primary health care, qualitative research methods

## Abstract

Iron and folic acid deficiency is a significant public health concern, especially in low and middle‐income countries, particularly among women of reproductive age, leading to adverse maternal and child health outcomes. In Pakistan, approximately 41.7% of women of reproductive age have iron deficiency. For this research, we conducted 33 key informant interviews and 32 focus group discussions with stakeholders from all four provinces and the two federally administered regions of Pakistan for in‐depth and representative bottleneck assessment. The modified Tanahashi model was used to inform the determinant codes and for coding reliability thematic analysis using NVivo 14. On the supply side, barriers relate to effective forecasting, procurement, and distribution of Iron Folic Acid supplements to the recipients. The demand–supply gap widened due to the supply bottlenecks, impacting the community's trust in the frontline health worker. These factors were gravely impacted by the governance challenges of ensuring sustainable funds, minimizing bureaucratic and procedural delays, and eliminating political pressure. Affordability of Iron Folic Acid and conservative social norms influenced the demand and awareness of Iron Folic Acid and nutritional knowledge among the target population. The stakeholder recommendations emphasize the need for intersectoral collaboration, mass media campaigns, frequent capacity building and incentivizing of healthcare workers, engagement of key community stakeholders, digitizing the health system, improvement in supply chain mechanism, and investment in small‐scale innovative solutions.

## Summary

- Trust in lady health workers (LHWs) is inadvertently compromised due to IFA stockouts and procurement delays based on population demands, significantly affecting the acceptability, uptake, and adherence to IFA supplements.
- Patriarchal norms and male‐dominated decision‐making affect women's access to IFA, whereby counseling sessions are often perceived as ineffective.
- Inadequate motivation, resources, and supportive supervision hinder effective coverage in LHW‐covered areas, while nutritional activities in uncovered areas suffer from inconsistent funding and lack of a legislative framework.
- Bureaucratic and funding challenges, along with existing policies, impact the tracking, registration, and supplementation of these communities' nutritional needs.

## Graphical abstract

Iron and folic acid deficiency is a significant public health concern, especially in low and middle‐income countries, particularly among women of reproductive age, leading to adverse maternal and child health outcomes. The modified Tanahashi model was used to inform the determinant codes and for coding reliability thematic analysis using NVivo 14. On the supply side, barriers relate to effective forecasting, procurement, and distribution of Iron Folic Acid supplements to the recipients. The demand–supply gap widened due to the supply bottlenecks, impacting the community's trust in the frontline health worker.

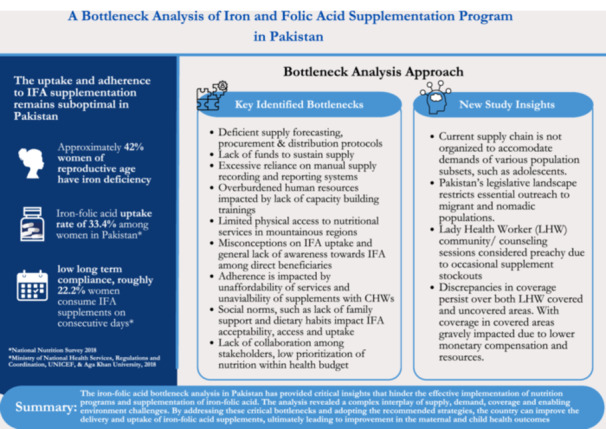

## 1 Introduction

Anemia is defined as low hemoglobin levels or poor quality of red blood cells, resulting in an insufficient and inadequate supply of oxygen to bodily tissues. Iron deficiency anemia (IDA) is primarily caused by poor nutrition, often due to imbalanced diets, cultural dietary restrictions, and limited iron‐rich food sources. During pregnancy, IDA poses substantial health risks to both the mother and developing fetus, increasing the likelihood of preterm birth, low birth weight, and maternal mortality (Baig‐Ansari et al. 2008). Similarly, pregnant women having IDA are susceptible to fatigue, weakness, and decreased immune function, making them more vulnerable to infections. Overall, IDA has a direct impact on neonatal mortality (1.8%) and infant mortality (2.9%). Furthermore, children born to mothers with IDA are at risk of inheriting the condition, perpetuating intergenerational cycles of poor maternal and child nutrition health. In infants and young children, IDA can hinder growth and cognitive development and weaken the immune system, making them more susceptible to infections. Severe cases of anemia in children can lead to developmental delays, increased morbidity, and even mortality (Abu‐Ouf and Jan 2015).

Globally, 37% of pregnant women, 30% of women 15–49 years of age, and 40% of children 6–59 months (about 5 years) of age are anemic (World Health Organization 2023). It is particularly concentrated in low and middle‐income countries (LMICs). Since the year 2000, there has been insignificant change in the worldwide prevalence of anemia among women of reproductive age (WRA), while the prevalence of anemia in pregnant women has experienced a minor decline (World Health Organization 2021). Recent studies have shown that the prevalence of anemia in LMICs has reached almost 60%. In Pakistan, there is a high burden of anemia in rural areas, whereby adolescent girls in rural areas are more likely (56.0%) to be anemic than their counterparts in urban areas (52.5%). Similarly, about 41.7% of WRA are anemic, with a slightly higher proportion in rural (44.3%) than in urban settings (40.2%) (Ministry of National Health Services, Regulations and Coordination, United Nations Children's Fund UNICEF, and Aga Khan University 2018).

In Pakistan, the province of Sindh has the highest prevalence of IDA, affecting 22.5% of WRA and 27.6% of pregnant women. Balochistan and Punjab have slightly lower rates. Khyber Pakhtunkhwa shows the lowest prevalence, with 15% among WRA and 15.3% among pregnant women. Federally administered regions like Azad Jammu and Kashmir (AJK) and Gilgit Baltistan (GB) have rates that are closer to the national average (Ministry of National Health Services, Regulations and Coordination, United Nations Children's Fund UNICEF, and Aga Khan University 2018).

Addressing IDA is crucial for reducing maternal and child mortality and morbidity. Implementing iron supplementation programs, promoting iron‐rich diets, and improving access to antenatal care (ANC) and maternal health services can play a significant role in preventing and treating anemia (Sanghvi, Harvey, and Wainwright 2010). Worldwide, governments are investing in IFA programs to reduce mortality rates and prevent lifelong health risks (Meier et al. 2003). In the past decade, the Government of Pakistan has implemented similar programs. Iron and folic acid (IFA) supplementation is an integral part of policies and programs implemented by Pakistan's provincial governments to combat perinatal complications, improve nutrition, and address IDA. These programs include ANC services provided through primary health care outpatient facilities and community outreach via community health workers, most notably the Lady Health Worker (LHW) Programme (“IFA Study Pakistan Report,” n.d.). ANC services prioritize safe pregnancies, including nutritional support with IFA supplementation, which play a pivotal role.

Despite extensive efforts and policies, the uptake and adherence to IFA supplementation remains suboptimal. Pakistan's National Nutrition Survey (NNS) 2018 reports a national uptake rate of 33.4% for women. IFA supplementation uptake rates are highest in Gilgit Baltistan (54.2%) and lowest in Balochistan (14.2%). In terms of compliance with the recommended daily intake of IFA, the statistics reveal more positive results, with 65% of women nationally adhering to the recommended daily dose, with the highest compliance in Gilgit‐Baltistan (81.4%) and the lowest in Balochistan (47.3%). However, long‐term compliance is notably low, with only 22.2% of women consistently taking IFA supplements for the recommended consecutive days (Ministry of National Health Services, Regulations and Coordination, United Nations Children's Fund UNICEF, and Aga Khan University 2018).

This IFA bottleneck analysis aims to improve the understanding of challenges in Pakistan's IFA supplementation program with a view to inform programming, planning, and implementation to achieve greater coverage and sustain quality. This analysis considers both supply and demand‐related factors to identify the most pervasive challenges to service delivery and reasons for low uptake.

## 2 Methods

### 2.1 Study Setting, Design, and Sampling

This qualitative study involved key informant interviews (KIIs) and focus group discussions with direct and indirect nutrition program beneficiaries from selected districts in the provinces and regions of Pakistan (including, Sindh, Baluchistan, Punjab, Khyber Pakhtunkhwa, Gilgit‐Baltistan, and Azad Jammu Kashmir). The range of target areas was set to capture geographical and provincial variations in IFA supplementation and bottleneck analysis.

The district selection was informed using the National Nutrition Survey (NNS) and other relevant gray literature. The following criteria were applied in district selection: (i) medium IFA supplementation coverage, (ii) medium population density, (iii) a balanced urban‐rural population ratio, and (iv) equal representation of southern and northern ethnic groups of the province. The following districts included Chiniot and Multan from Punjab, Charsadda and Swat from KP, Muzaffarabad from AJK, Sanghar from Sindh, Ziarat from Baluchistan, and Gilgit from GB. Few districts were targeted for a deeper assessment of bottlenecks. Low‐coverage districts were excluded to enhance the assessment and to optimize the identification of bottlenecks. KII participants were purposively sampled in consultation with the Provincial stakeholders (Department of Health), the nutrition wing of MoNHSRC, and UNICEF Pakistan. The nomination process aimed for a balanced representation of service delivery stakeholders, including decision‐makers and implementors. A total of 33 KIIs were conducted with Director General Health Services (DGHS) (*n* = 3), Director of Nutrition (*n* = 6), Provincial Coordinator of the LHW Programme (*n* = 6), Female Medical Officers (FMO) (*n* = 8), District Health Officers (DHO) (*n* = 8), and District Coordinator of the LHW Program (*n* = 8) (Table 1).

**Table 1:** Number of Key Informant Interviews (KIIs) by key stakeholder group and study district in each province or region in the bottleneck analysis in Pakistan.

DG health service			Director nutrition	Provincial coordinator of the LHW program	District health officers (DHO)	District coordinator of the LHW program	Female medical officers (FMOs)	Total
**Province/Region**	**District**							
Punjab	Chiniot	0	1	0	0	1	1	3
	Multan	0	0	0	0	1	1	2
Khyber Pakhtunkhwa	Charsadda	1	1	1	1	1	1	6
	Swat	0	0	0	1	1	1	3
Sindh	Sanghar	1	1	0	1	1	1	5
Baluchistan	Ziarat	0	1	1	1	1	1	5
AJK	Muzaffarabad	1	1	1	1	1	5	10
GB	Gilgit	0	1	1	1	1	4	9
	Total	3	6	4	12	8	33	

### 2.2 Data Collection

The KIIs aimed to assess stakeholders' awareness and their understanding of nutritional deficiencies, their knowledge of nutrition programs, including the IFA supplementation program, and their roles and responsibilities. The interviews also explore the barriers and facilitators in IFA service delivery (Annexes A–C). Although all participants agreed to participate, representation was impacted by institutional challenges i.e., in some cases, interview respondents were handling additional charges from two different offices.

Focus Group Session (FGD) participants were purposively sampled. The participants include frontline health workers, i.e., LHWs, lady health visitors (LHVs), as well as direct beneficiaries of IFA supplementation, i.e., pregnant and lactating women (PLWs) and fathers. The LHWs (*n* = 8) and lady health visitors (*n* = 8) were nominated by the provincial health departments. The nominated LHWs recruited fathers (*n* = 8) and PLWs (*n* = 8) for the FGDs based on preset selection criteria (i.e., residents living in the district for at least 6 months, mothers who are currently pregnant or lactating, and husbands of PLWs women or fathers). The discussions aimed to identify factors that may influence the likelihood of IFA uptake, as well as the forms and sources of information that respondents rely on to make health‐related decisions (Annexes D–F). A total of 32 focus group discussions were conducted, with 4–6 participants per category (Table 2). Although all participants agreed to participate, participation was impacted by scheduling challenges.

**Table 2:** Number of Focus Group Discussions (FGDs) by stakeholder group and study district in each province or region in the bottleneck analysis in Pakistan.

Lady health workers (LHWs)			Lady health visitors (LHVs)	Pregnant and lactating women (PLW)	Fathers/Husbands of PLW	Total
**Province/Regions**	**District**					
Punjab	Chiniot	1	1	1	1	4
	Multan	1	1	1	1	4
Khyber Pakhtunkhwa	Charsadda	1	1	1	1	4
	Swat	1	1	1	1	4
Sindh		1	1	1	1	4
Baluchistan		1	1	1	1	4
AJK		1	1	1	1	4
GB		1	1	1	1	4
	Total	8	8	8	8	32

### 2.3 Data Analysis

Monitoring Results for Equity Systems (MoRES) was employed to pinpoint and emphasize significant obstacles within Pakistan's IFA supplementation program. The MoRES approach is useful in assessing interventions/programs to address inequities and barriers that impede the achievement of desired outcomes by using a determinant framework and analyzing the main causes of existing bottlenecks.

#### 2.3.1 Tanahashi Model

The determinant framework for qualitative analysis was derived from a review of previous studies on bottleneck analysis conducted in the LMICs. The *Modified Tanahashi model* was adopted to identify bottlenecks in the service delivery domains among the target population. The modified model introduces the ‘*enabling environment*’ domain in addition to the *demand*, *supply*, and *effective coverage* domains (O'Connell and Sharkey 2013; Berg and Lune 2012; Downe‐Wamboldt 1992). The enabling environment domain consists of determinants of social norms to ensure that factors related to societal practices, policies, legislation, governance, and budgets are covered when identifying bottlenecks. The study defines supply‐related determinants as the factors that influence health care's ‘production function’. These factors include the availability of essential health commodities, the availability of human resources, and accessibility to services. Demand‐related determinants include initial utilization and continuous utilization of a service, existing at the community, household, and individual levels, and are influenced by demand. Effective coverage is defined as coverage of sufficient quality to reach a defined health impact (Wing 2018).

The study only deals with the qualitative aspects of the Tanahashi framework and, therefore, employs the use of NVivo 14 software to derive themes and subthemes from the study while limiting research bias. A dedicated team of six researchers was trained to analyze data using NVivo 14, led by a research lead. Each researcher was randomly paired to peer‐review codes to ensure intercoder reliability and to reduce the bias of having one individual coder decide upon themes. These researchers were not involved in the data collection process, further enhancing study rigor. The lead researcher, informed by the determinant framework, merged, reviewed, and prepared the final codebook (Annexes G and H). The final codebook reflects the rigorous and iterative process of data analysis and categorization within the four *Tanashashi* service delivery domains. The process allowed the team of qualitative analysts to identify bottlenecks at all levels—the government level, the level of health care providers (HCPs), and the community level.

The KIIs and the FGDs were conducted by seasoned research experts and trained field staff, respectively, in Urdu and in person. The interview and focus group guides were piloted/pre‐tested before data collection activities commenced. This pre‐testing process helped to refine questions and ensured sensitivity for the respondents. The senior research team trained the field team to conduct FGDs.

All participants were provided with a consent form (Annex I) explaining the study aim, associated risks, and confidentiality provisions. Signed consent was obtained from all study participants. The proceedings from the interviews and discussions were digitally recorded with permission, transcribed verbatim, and then translated into English. Any notes taken during the interview process were consolidated to aid data completeness and comprehensiveness.

### 2.4 Ethical statement

Approval for this study was obtained from the Research Ethics Committee of the National Bioethics Committee (NBC) Pakistan (ref. No.4‐87/NBC‐488/20/XXX).

## 3 Results

The findings are presented in the following four sections: (1) Supply‐side factors, including the availability of essential commodities, availability of human resources, implementation service delivery, and accessibility of services; (2) Demand‐side factors, including acceptability and affordability of services upon initial utilization; (3) Coverage of services, including the quality of coverage in terms of its adequateness, and (4) Enabling environment, including the social norms, policies and legislation, budget or expenditure and governance barriers. The respective subthemes are explored in all sections. The codes and quotes are attached in Annexes G and H.

### 3.1 Supply‐Side Factors

The reliance on manual systems and the bureaucratic structures impacts timely IFA procurement, in the event of a stockout. The late supply procurement begets a supply gap due to procedural delays. As a result, there is a trickle‐down effect on commodity distribution, which is insufficient to meet the population's demand.

#### 3.1.1 Availability of Essential Commodities

The reported availability of IFA supplements across regions is marked with critical uncertainty, inconsistency, and disparity. There are discordant reports of commodity availability from the fathers, PLW, and LHWs. Only some of the fathers reported that IFA supplements are adequately available at the LHW or the facility pharmacy. However, most LHWs and other key stakeholders report a shortage of IFA and concerns about the quality of medicine available in the market. PLWs are often prescribed and provided either iron or folic acid, whichever is available.

Mostly women tell us that there is a lot of difference between the tablets they take from the market and the tablets that we provide them. They say those tablets that they purchased from the market are less effective.

(KII_LHW)

##### 3.1.1.1 Deficient Supply Distribution, Procurement, and Forecasting Mechanism

The LHWs share the stock with BHUs/RHCs, to support their daily activities. The majority of LHWs report that supply distribution and procurement depends on the patient load attended by the health facility during the previous month, irrespective of the type of healthcare provider and their community presence. Buffer stocks are not maintained. Most BHUs receive the IFA supply, which is sufficient to last 3 months only and may not effectively address the demand for the medicine, given each PLW requires at least 3 months' supply. Therefore, the supply forecasting mechanism is deficient. The procured stock is only sufficient to meet the demand of IFA by PLW, with the exclusion of other key populations.

Whatever we quote, it depends on the clientele. Last month, we had 200 ANC with frequent revisits. We gave three during PNC and then three more visits and then there are either BD doses or OD dose so because it fluctuates, sometimes we have it sometimes we run out we don't determine a fix amount to procure.

(KII_ BHU LMO_Sanghar)

##### 3.1.1.2 Lack of Funds to Sustain Supply

The majority reported a lack of funds to sustain supply, resulting in periods of IFA shortages. Most DHOs indicated that, since the PC‐1 protocols mandate the district office to procure the medicines on behalf of the health care facilities, the lack of funds at the district level affects the timely procurement of IFA. Additionaly provincial efforts to cover IFA demand for LHW uncovered areas have also been impacted due to lack of funds.

We thought we would ensure provision in areas where LHWs are completely absent i.e. uncovered areas, as identified by our DHOs but we were not given funds.

(KII_LHW_Baluchistan)

##### 3.1.1.3 Reliance on Manual Supply System

In all study areas, there is a high dependence on manual registers or record keeping at the facility level. The medical officer monitors and seeks monthly updates from the lady health supervisor, compounder, and storekeeper about the monthly stock turnover using manual stock registers.

This is the manual system. This system will run approximately for 2 years because we have large registers so, they will last for 2 years. We often face the issues in keeping records this way.

(KII_District Cordinator_Baluchistan)

##### 3.1.1.4 Provider Dependence on External Supply and Procurement Chains

Most frontline workers rely on non‐facility‐based pharmacies or medicine stores for PLW to have continued access to IFA supplements. The providers redirect the PLW to any pharmacy without confirming the availability of IFA. Additionally, some stakeholders report that the private sector engagement in IFA supplementation is limited in reach among most provinces, due to poor infrastructural capacity, especially in politically neglected regions, and high supplement cost. The dependence on external agencies or companies also extends to ensuring the availability of iron‐fortified foods, such as wheat and flour.

In Gilgit and Baluchistan, the tablet is not available so the LHWs, doctors and LHVs, prescribe them to take it from the market if it is available.

(KII_LMO_KP)

##### 3.1.1.5 Supply Stockouts

LHVs and LHWs FGDs reveal occasional stockouts of IFA in their practice, with significant shortages reported with LHWs in Baluchistan, Punjab, AJK, and GB region, sometimes lasting over 6 months. Both PLW and high‐level stakeholders confirm these systemic supply shortages. Additionally, inadequate disaster preparedness and climate‐resilient infrastructure divert supplies to affected communities, further depleting routine channels.

The quantity is not enough. The national program provides us with supplements but for the last 4 to 5 months we don't have medicines. Before 5 months, the supply of medicines was excellent but for 6 to 7 months and in between there is no medicine.

(KII_District Coordinator_Baluchistan)

#### 3.1.2 Availability of Human Resources

##### 3.1.2.1 Overburdened Human Resource

Most of the frontline workers and high‐level stakeholders are overburdened, handling multiple tasks that should be managed by dedicated offices. In some districts, especially in the LHW‐covered areas, the number of human resources is insufficient to meet the nutritional needs of the population. Most LHVs report similar findings. The patient load is also unevenly distributed, with some LHWs catering to only 20 families while others manage sixfolds in the patient load.

The major issue in Gilgit is HR. Any program which is introduced, though there is enough money, there is no HR. The medical super attendants, directors or DHOs are given an additional charge for daily operations. [Additionally] due to the absence of any vertical program the burden of work is felt at the gross root level by LHW.

(KII_Director Planning_GB)

##### 3.1.2.2 Lack of Motivation

Most frontline workers express dissatisfaction with financial remuneration and lack of resources. They often spend their own money on support materials, like work diaries. The work burden leads to frequent burnout, affecting LHWs' well‐being and efficiency, sometimes resulting in extreme cases such as reported death.

One of the workers died on her duty for 3rd polio vaccination. From Muzaffarabad to Raj Pindian, everybody was testifying to her intense work as a vaccinator and health educator in the community. I think we should lessen their burden.

(KII_Program Coordinator LHW_AJK)

##### 3.1.2.3 Lack of Frequent Capacity‐Building Training

At most healthcare levels, there is a lack of frequent capacity‐building or refresher training on healthcare providers' roles and responsibilities in IFA supplementation. In the case of training, the workload and poor training notifications prevent participation in training. Additionally, in most Basic Health Units (BHUs)/Rural Health Centers (RHCs), untrained storekeepers handle IFA stock maintenance, leading to potential discrepancies in IFA knowledge and practice.

I think they received training 2 or 3 years ago, maybe four. Everything is getting advanced and how would they know if they didn't get any training.

(KII_District Coordinator National Program_Baluchistan)

##### 3.1.2.4 Inadequate Supportive Supervision Support and Services

Most frontline workers report that the impact of nutritional services is affected due to subpar supportive supervision. Additionally, some nutrition program officers report that the widescale absence of dedicated technical support to design health communication is reported to impact the quality of IFA messaging. There is a reported absence of anemia testing services at some local health facilities.

Proper monitoring and supervision are needed for better performance.

(KII_Director Nutrition_GB)

##### 3.1.2.5 Quality of HCP Counseling Services

Most PLWs and fathers report that HCPs effectively counsel on the importance and method of IFA intake (e.g., at night, before meals, with milk or water, and not with tea). However, some express concerns over the comprehensiveness of the counseling sessions due to the healthcare provider's workload. Competing healthcare responsibilities, limited resources, and administrative challenges make it difficult for most LHWs to counsel effectively on all health topics. The knowledge of LHWs is often compromised due to inadequate education and training, leading to inconsistent advice on IFA consumption. Most counseling sessions tend to focus on the method of consumption and prevention of side effects but neglect side effect management.

In our provisional setup, we provide counseling for family planning and minor elements, such as nutrition deficiency, IDA or worm infestation. We prioritize the topical session which has more disease prevalence in the area so that more time is spent on that.

(KII_DHO_GB)

Mostly they don't know what IFA is. They will only know if the LHWs are educated. There are lady health workers who are too old like 50 years, and they don't have metric degrees even.

(FGD_Fathers)

#### 3.1.3 Accessibility of Services

##### 3.1.3.1 Physical Access to Services

Consumers often visit LHWs at their homes to obtain IFA supplements due to their strong community presence. However, access to health services and actors is limited in mountainous regions, especially for marginalized communities. In some areas, long travel distances and inadequate transportation further hinder access to health facilities and LHWs. In tribal areas, patriarchal values restrict LHWs’ ability to reach female household members.

There are few limitations as it's a tribal area, you cannot communicate with the women directly. You can only reach them though their husband, brother or son. Though LHW program is beneficial, you need to struggle and work.

(KII_Head of Health Department_Baluchistan)

##### 3.1.3.2 Factors Affecting Food Availability

Due to the poor availability of IFA supplements at health facilities and community levels, PLWs are alternatively counseled to consume nutrient‐rich foods to address nutritional deficiencies. In areas with difficult geographical terrain, access to the markets is limited, forcing PLW to rely on the food available at home. Furthermore, the poor implementation of food safety guidelines affects the quality of market food, deterring consumption of nutrient‐rich foods, such as those from fisheries in Gwadar, regardless of proximity to food sources.

Baaji (LHW) tells me to consume milk and fruits when she doesn't have medicine. We eat whatever is available at home.

(FGD 1 PLW)

### 3.2 Demand‐Side Factors

#### 3.2.1 Initial Utilization‐Acceptability

##### 3.2.1.1 Lack of Awareness Among Direct Beneficiaries

There is a general lack of awareness about IDA, IFA interventions, and the importance of a balanced diet. PLW prefer to eat traditional or cultural food easily made available. Certain superstitious beliefs also impact nutritional knowledge and the acceptability of nutritional interventions, such as associating shortness of breath with paranormal possession. The nutritional knowledge is also impacted by the literacy levels of the pregnant women.

They aren't aware of what a balanced diet for them even would be. They just eat according to their preference.

(KII_LMO_Sanghar)

##### 3.2.1.2 Misconceptions on IFA Uptake

There are societal concerns over the integrity of counseling sessions on IFA. Males do not trust sending their wives to these sessions as LHWs provide sessions on family planning. They think that IFA consumption may lead to miscarriage or infertility.

A lot of them don't take these tablets or give it to their children and stop contacting LHWs due to fear of miscarriage or infertility in their daughters or sons.

(FGD_PLW 6_AJK)

##### 3.2.1.3 Initial Use Concerns

The initial use concerns were associated with the taste, color, appearance, IFA dosage, medicine shelf life and the side effects. Table 3 explores the specific concerns with IFA initial utilization.

The only complaints we usually receive from the community are that continuous intake of IFA disturbs the stomach, and if you consume it for more than 10 days, it affects gastric motility as it causes constipation. Sometimes people report complaints about loose stools or black stools. There are frequent complaints.

(KII_PD Nutrition Program_Punjab)

**Table 3:** Initial utilization concerns with IFA consumption.

Initial use concerns	
Concerns with taste, color, and appearance	There are concerns over the taste and color of the IFA supplements, due to which PLW prefers to have fortified foods over IFA supplements. There are also concerns about the IFA bottle's appearance, which causes suspicion about its purpose and probable linkages with family planning.
Concerns with IFA dosage	LMOs raise concerns over the dosage of IFA, which may lead to lower compliance.
Concerns with stomach Issues	PLWs raise concerns over stomach issues and diarrhea due to the continuous intake of folic acid, which prevents them from consuming IFA.
Concerns with medicine shelf life	Community members have noted that the medicines provided by healthcare providers may often be close to expiry due to poor stock turnover. Similar concerns with expiry are noted at the district level.

##### 3.2.1.4 Low Adherence Among Users

Adherence among LHWs is severely impacted by supply‐side barriers, leading to an implicit lack of trust when LHWs fail to provide IFA supplements as expected. Communities expect LHWs to supply IFA, given their advocacy for its use during pregnancy. Discontinuation is also influenced by low literacy and education levels among PLWs, who may not fully understand the increased nutritional needs during pregnancy.

Inadequate awareness of IFA interventions among PLWs further affects adherence. Cultural food norms, such as tea consumption, interfere with proper IFA absorption. Awareness inequities exist between family members and PLW in LHW‐covered versus uncovered areas, leading to inconsistent adherence to IFA courses to prevent or treat anemia.

The free provision of IFA supplements generally facilitates consumption and adherence, though some perceive free supplements as low quality—a belief held by both high‐level stakeholders and grassroots communities.

Our girls were fearing that community demand from them, and when we are empty‐handed, we just talk, and everyone gives advice. People get fed up with verbal things, they will say either give us the money or IFA tablet. We are losing out trust due to no availability.

(KII_Program Coordinator LHW_AJK)

##### 3.2.1.5 Lack of Targeted IFA Communication

The IFA messaging is not tailored to address the specific needs of specific populations or ethnic subsets according to the cultural food practices of the communities. These factors impact the acceptability of IFA among the population.

What we do is that we generalize our messages, and we think that our message will address all the issues for all the population in the same way. This is not the right way of doing things. We should know their food routines are, the type of food they consume and understand if there is a specific issue lying in the food habit in specific area. Then we design a targeted campaign to change their food habits.

(KII_PD Nutrition Program_Punjab)

#### 3.2.2 Initial Utilization‐Affordability

##### 3.2.2.1 Lack of Affordability

The affordability of IFA supplements is a significant concern for vulnerable populations experiencing poverty and food insecurity. Shortages of facilities and LHWs force reliance on external supply chains, and the cost of IFA in the market impacts access and compliance for financially vulnerable communities. This dissatisfaction leads to negative perceptions about the medicine's effectiveness.

Socioeconomic issues are a primary cause of maternal malnutrition in various districts. Lack of well‐paying jobs reduces families’ purchasing power, increasing their reliance on local food sources, which poses challenges in terms of availability, quality, and cultural dietary norms. Additionally, families often sell their best produce to earn money rather than consuming it themselves, reflecting the socioeconomic struggles that lead to compromised nutrition.

It cost RS. 50 almost and it contains 100 tablets. And there are people who can't afford even this.

(FGD_LHV_AJK)

### 3.3 Coverage of Services

#### 3.3.1 Quality of Coverage

##### 3.3.1.1 Differential Access to Services

The LHW coverage for certain populations, such as the nomadic community is deficient due to the national program policy, which mandates the provision of health services only to residents who have lived in an area for a certain time. The difficult terrain also raises access issues for these communities due to the distance from the main road. Additionally, even in the LHW‐covered areas, the number of staff is inadequate due to high worker retirement rates and low enrollment rates in the LHW program.

Because most of our community do not have the road access as well, so sometimes it is difficult to reach them.

(KII_DHO/DC_Multan_Punjab)

##### 3.3.1.2 Unstable Coverage of Services

In LHW uncovered areas, the coverage of services is deficient, marked with interruptions and lack of consistency. These areas are often neglected due to the lack of resources to access these areas. Due to the unstable coverage, the quality of IFA awareness varies. Additionally, where covered areas may get exposure to LHW mass awareness sessions, the population residing in the uncovered areas do not receive exposure to community health education activities, such as “LHW Nutrition Week,” which further limits their exposure to communication on maternal nutrition.

Where there LHW does not visit, the women there even cannot visit hospitals. They don't know about the diet. They have no education and knowledge about it.

(KII_FGD_LHW_AJK)

### 3.4 Enabling Environment

#### 3.4.1 Social Norms

##### 3.4.1.1 Lack of Family Support

The lack of family support for IFA impacts the acceptability, accessibility, and consumption of the medicine. Due to a lack of awareness, mothers‐in‐law or husbands are not allowed to purchase and consume IFA. This ignorance about the increased nutritional needs during pregnancy is also prompted by traditional beliefs and a reluctance to engage in sessions due to competing household responsibilities.

Often women visit me and start crying that her mother‐in‐law doesn't allow to consume IFA.

(KII_WMO_Chiniot_Punjab)

##### 3.4.1.2 Health Seeking Behavior

Most pregnant women reported visiting the doctor for their first ANC checkup in the 2nd or 3rd month of pregnancy; however, there are accounts of PW who were 5 or 6 months pregnant late for their first ANC checkup. People also tend not to show up for follow‐up visits. The lack of availability of IFA with the health workers and the health‐seeking behavior of the PLW higlights the gaps bewteen supply and demand. When the supply depletes, PLW shows greater preference for obtaining IFA from the LHW rather than the market. In most cases, they wait for the supply to be restored by the LHW.

The fertility rate here is more than 2.5 so due to this there is a gap [to follow the] recommended birth spacing. It is recommended that after bearing one child, females prepare her body for the next childbirth with the help of supplementation, as the stores of iron deplete in them. If you will not prepare her body for the next childbirth, no doubt she will continue being iron deficient. They give birth to 4 to 5 children and may eat fortified.

(KII_Director Planning_GB)

##### 3.4.1.3 Internalized Social Norms

The internalized social norms relate to dietary habits or patterns, the patriarchal mindset and the gendered aspect of food norms. Table 4 explores the social norms which impede IFA access and utilization.

Here females are financially dependent on males. Men should also be involved because they often think that females are telling lies. Females feel that their husbands do not trust them; they often bring them here as well. They ask us to counsel them.

(KII_LMO_Multan_Punjab)

**Table 4:** Social norms affecting the nutritional status of pregnant women.

Internalized Social Norms affecting nutritional status of pregnant women	
*Dietary Habits and Patterns*	In most places, the internalized gender sociocultural norms dictate that women eat last in the household. Most frontline workers reported that the norms limit adolescent girls' consumption of nutrient‐rich foods due to beliefs that these foods may trigger early aging or worsen obesity, while men need energy‐dense food to support their families. LHVs report that pregnant women sometimes avoid perceived iron‐rich foods like poultry due to food taboos, despite counseling.
Patriarchal norms	In some areas the husbands do not trust their wives to make decisions independently, as a result financial disempowered PLW accompanied their husbands to ANC sessions, so that the husband could witness the counseling.
Prioritization of male children	It is reported that the focus on ensuring healthy diet is directed more toward male children rather than female children because of the perception that males will financially support the family and need physical strength. In these areas, male children continue this norm, having observed it in their homes while growing up.

#### 3.4.2 Policies and Legislation

##### 3.4.2.1 18th Amendment and PC‐1

Most district officers report that institutional changes, like the 18th Amendment, have increased vulnerability to supply chain disruptions by deprioritizing funding for nutrition programs. Developing and implementing PC‐1 is viewed as an effective solution to these challenges. However, delays in fund release due to PC‐1 expiry and subsequent approvals cause procedural and procurement delays. Additionally, the roles of actors frequently change based on the approval of PC‐1. These issues are particularly evident in AJK, GB, and Baluchistan.

There are many financial crunches after the 18th amendment where social sector development is least prioritized.

(KII_Program Coordinator Nutrition_AJK)

##### 3.4.2.2 Existing Nutrition Intervention and Practices

Nutrition programs in Pakistan are often short‐lived or suspended due to budgetary or administrative constraints and in some areas they do not exist at all. These programs are typically run at the behest of international donors in limited districts for a limited time. Moreover, programmatic efforts are primarily focused on children rather than mothers or adolescents. This results in a clear gap in addressing maternal malnutrition compared to child's nutrition, as exemplified by programs like the OTP (Outpatient Therapeutic Program for SAM in children).

We have children under [sic] 5 years, we conduct a MUAC, check their height and weight. If they are suffering from malnutrition we refer them to OTP. For mothers there isn't anything of that sort, so we just give them iron folic acid.

(KII_LMO_Sanghar_Sindh)

##### 3.4.2.3 Insufficient Use of Various Modes of Communication

Reliance on LHW community mobilization activities is critical for conveying maternal nutrition and IFA supplementation messages in most provinces. Other media forms are less relied upon, especially in culturally conservative areas like tribal regions of Baluchistan, where female mobile ownership is limited, making LHW sessions the optimal approach. While print and electronic media are used to communicate nutrition messages, their use is limited despite their potential for greater outreach. The current modes of communication also lack reach due to the lack of translation in local languages. Additionally, the technical skills required to design proper awareness campaigns are deficient, which leads to unimpactful messaging.

In this important massage should be advertised in local language. Inform in local language what happens with iron and folic acid deficiency.

(KII_Director Nutrition_GB)

##### 3.4.2.4 Other Policies and Legislation Impacting Access

The health systems mandate that the provision of health services must be ensured to the community members who stay or reside in a place for at least 6 months. The legislation heightens the health access challenges to nutritional services for hard‐to‐reach nomad and migrant population. This becomes a critical administrative challenge due to registering and following up with the population. There is a subsequent lack of adequate staffing to address the health concerns of these populations.

One thing is, we include in our register people who stay somewhere for at least six months, some people settle in a place only for five to ten days, or for a month, if we register them, it becomes difficult for us to follow them.

(KII_DHO_GB)

#### 3.4.3 Budget or Expenditures

There are marked budgetary constraints in supporting nutrition program efforts. Ineffective budget planning leads to fund shortages and unpreparedness in the event of an unforeseen circumstance, such as a disaster or climate catastrophe. There is subsequent pressure on government to reduce their nutritional budget requested in the PC‐1s. There is also limited funding for nutrition programs to address nutrition challenges due to the short‐term nature of programs and dependence on external funding.

Nutrition is an expensive business. Its cost was so high, and the government said that our development budget is equal to your nutritional budget and asked you to reduce it.

(KII_LHW Cordinator_Baluchistan)

#### 3.4.4 Governance

##### 3.4.4.1 Inadequate Infrastructural Support and Disaster Preparedness

The absence of climate‐resilient infrastructure poses significant challenges. Pregnant women report that before the pandemic, LHWs and health facilities provided free IFA supplements, typically lasting for 3 months. However, during disasters, resources are diverted from nutrition services to immediate health responses, compromising adequate nutrition support. Inadequate infrastructural support prevents many LHWs from operating health houses, forcing them to rely on securing space in pregnant women's homes due to space constraints.

All nutrition sites we had were closed and remaining stabilization centers were converted to COVID isolation wards.

This is strange that it is not taken as a priority by us, i.e. malnutrition is taken as something at the bottom and considered as the last thing. It's not serious.

(KII_LHW Cordinator_Baluchistan)

##### 3.4.4.2 Lack of Collaboration Between Public and Private Sector

There is non‐strategic engagement with the private sector, with subsequent reluctance to collaborate with the private sector due to duplication of efforts which is believed to impact the demand for the public sector's services.

I think, it is good (to be done) at the urban level, but sometimes this causes doubling. we have such experience at one or two places, if they are involved, our work gets effected.

(KII_DHO_GB)

##### 3.4.4.3 Inefficient Utilization of Information Systems

Logistic management information system (LMIS) and vaccine logisitic management information system (vLMIS) are available, but that is used for other purposes, not IFA. The IFA logistics is mostly maintained manually through LHW registers and shop keepers’ registers at the facility level. Mostly digitization of health records is carried out at the district level. The staff is also not trained to use computer systems to enter logistic information regarding the existing supply of IFA at the primary healthcare level.

We have LMIS and vLMIS, but it is not specific to these iron Folic acid supplements. They have their own way of managing which is normally done manually.

(KII_PD Nutrition Program_Punjab)

## 4 Discussion

The effective implementation of IFA supplementation has proved challenging in many lower middle‐income countries, particularly in terms of coverage and compliance. Previously, similar bottleneck analyses on IFA service delivery were conducted in Africa and South Asia, which revealed a similar landscape for IFA service delivery. Accessibility in terms of logistics is a persistent challenge to IFA supplementation in lower middle‐income countries in general and in South Asia specifically (Gilson and Raphaely 2008; Ogbo et al. 2019; Wilson et al. 2013). A study conducted in India in 2019 identified inconsistent supply as a barrier to IFA supplementation programs, affecting both supply and demand. Pregnant women reported receiving supplements, while those who did receive them often didn't comply with recommended intake guidelines due to the belief that supplementation was unnecessary (Varghese et al. 2019). Logistic barriers and inaccessibility were also identified as concerns in some villages. The issue with the gaps between supply and demand was also observed within the IFA supplementation program in Pakistan. The results of this assessment indicated that due to inconsistent and inadequate supply, there are frequent stockouts, often lasting between 3 and 6 months at the primary healthcare level. It was reported that even when stocks are available, district‐level provisions are only sufficient to meet 50% of the demand. Additionally, it was also found that due to the lack of frequent trainings (on nutritional counseling and communication) of the health workers, especially the LHWs, they are unable to effectively communicate information on IFA supplementation to communities, including information on dosage, compliance, and potential side effects. The lack of and/or undertrained frontline health workers contributes to maternal mortality, as evidenced by a study conducted in Pakistan on midwifery education and maternal and neonatal health issues which uncovered that improving the skill and knowledge levels of health staff, especially midwives and nurses can reduce maternal mortality rates (Rukanuddin, Ali, and McManis 2007). Therefore, addressing supply issues and misconceptions and improving accessibility are crucial for effective implementation. Although an inadequate supply of IFA supplements adds to the low uptake of the supplement, nutritional illiteracy also significantly contributes to it. In the context of Pakistan, it was reported that due to low awareness among pregnant women and their families regarding IDA and IFA supplementation; ensuring high uptake of the supplement remained a challenge. Similarly, due to low levels of awareness, pregnant women visiting ANC was also low. This is consistent with the evidence from Ethiopia, where healthcare providers reported an increasing demand by women attending ANC since the awareness regarding the supplement improved, hence enabling a higher IFA coverage (Siekmans et al. 2018). Another barrier adding to the demand‐side challenges was the community's concerns about the side effects of taking IFA supplements. Given that the healthcare workers do not effectively counsel pregnant women and their families on the management of potential side effects of the supplement, it was found that this contributed to low consumption rates at the pregnant women's end. The quality of counseling was also impacted due to an overburdened workforce, lacking motivation due to delays in salary release, and an imbalanced workload. This mirrors the situation in Nigeria and Ethiopia, where misconceptions regarding the side effects of IFA hindered women's willingness to take the supplement (Siekmans et al. 2018).

To determine the successful roll‐out of IFA supplementation, it is pertinent that a robust delivery system is in place. A study conducted in 2017 in Afghanistan, Bangladesh, Indonesia, Ethiopia, Kenya, Nigeria, and Senegal found that ANC services, including IFA supplementation, are adversely affected due to barriers in the delivery system (Siekmans et al. 2018). This is true for Pakistan as well, and this bottleneck analysis highlighted that the lack of logistical planning coupled with an inefficient logistics system hampers the supply and delivery of IFA across the country. This is most evident in hard‐to‐reach communities where weak infrastructure pronounces issues with a continuous supply of healthcare commodities. It is vital for countries to have up‐to‐date, accurate data available through their Health Management Information System (HMIS) and Logistic Management Information System (LMIS) to identify challenges to IFA service delivery. However, many countries lack such data. A study conducted across five Southeast Asian countries (Cambodia, Laos PDR, Thailand, Indonesia, and Vietnam) found that only one country (Thailand) has enough national data available on the situation on the ground. The lack of context‐specific data on IFA consumption and coverage is true for Pakistan as well. Through the bottleneck analysis, it was found that despite having the Vaccine Logistics Management Information System (VLMIS), IFA supplementation is not part of it whereby only manual records are kept on the supplementation. This lack of proper record‐keeping exacerbates the situation as it limits the timely communication between stockouts and stock management.

A bottleneck analysis conducted in Rwanda in 2017 identified barriers in maintaining good maternal and child health in the country. Despite a robust healthcare policy, the system failed due to the lack of implementation, which rested on the in adequate availability of healthcare personnel and health facilities (Khurmi et al. 2017). Like the findings from Rwanda, it was noted in Pakistan that the Department of Health struggles to fill human resource gaps, resulting in shortages of essential health workforce. Shortage of health staff, especially the LHWs, which adversely impacts the IFA program's coverage and reach. Given that much of Pakistan's territory is rural, it was found that rural areas were not adequately covered by LHWs. Resultantly, it was reported that rural populations in uncovered areas exhibit the lowest levels of knowledge regarding iron deficiency anemia (IDA), its consequences, IFA supplementation, balanced diets, and the importance of essential nutrients in preventing nutritional deficiencies. This lack of awareness contributes to limited understanding and poor adoption of preventive measures, including the uptake of appropriate supplementation. Addressing this knowledge gap is crucial to improving nutritional outcomes and reducing the prevalence of IDA in rural communities in Pakistan.

While structural barriers pose immense challenges to maternal and child health especially in lower middle‐income countries, cultural barriers are also problematic. Cultural barriers to accessing health care are difficult to address, as they are context‐specific and vary from country to country. Formative research conducted in seven countries of Africa and Asia summarized the barriers and enablers for improved coverage and utilization of IFA supplements by pregnant women. With respect to relational barriers, it was found that in Afghanistan, Senegal, and Nigeria, the decision‐making power typically rested with husbands or mothers‐in‐law who determined the pregnant woman's attendance at antenatal care (ANC). This was true for Pakistan as well, where most of the household decision‐making power lay with men and/or mothers‐in‐law. In Bangladesh and Nigeria, older adult women sometimes discouraged younger women from seeking ANC services and using medications during pregnancy. In a similar context, the pregnant women in Pakistan lacked agency in terms of making decisions that could help their nutritional status, and their diets were restricted by the mother‐in‐law based on her own beliefs around nutrition and diets and cultural food taboos.

### 4.1 Stakeholder Recommendations

Health officials recommend improving the supply and demand sides of IFA supplementation through several measures. On the supply side, recommendations include ensuring an uninterrupted supply of IFA, timely reporting of stock levels, and demand‐based distribution are essential. Enhancing the skills of healthcare workers through refresher training, seminars, increasing supervision and attendance monitoring are also recommended. Recruitment and incentivizing LHWs to cover remote areas, along with adequate funding and a unified national logistics system are crucial. On the demand side, community mobilization sessions, family counseling, and using media like radio programs to raise awareness about IFA among PLWs and their families are recommended as key strategies. Involving community stakeholders, such as religious institutions and local leaders is also important. To create an enabling environment, stakeholders recommend collaborating with the private sector, digitizing health systems, decentralizing authority, and promoting public health education and nutrition. Communication and media play a vital role in enhancing health programs, using creative strategies to reach remote areas effectively.

### 4.2 New Insights From the Study

The study offers the following new insights into bottleneck analysis for IFA supplementation in LMICs, particularly Pakistan.

i. Trust in LHWs is inadvertently compromised due to stockouts, significantly affecting the acceptability and adherence to IFA supplements, with counseling sessions often perceived as preachy.
ii. Discrepancies in coverage exist both within LHW‐covered areas and between covered and uncovered regions. Inadequate motivation, resources, and supportive supervision hinder effective coverage in LHW‐covered areas, while nutritional activities in uncovered areas suffer from inconsistent funding and lack of a legislative framework. Additionally, Pakistan's legislative landscape restricts essential outreach to nomadic and migrant populations, with physical access further hampered by difficult terrain.
iii. Bureaucratic and funding challenges along with existing policies, impact the tracking, registration, and supplementation of these communities’ nutritional needs. Furthermore, the current supply chain mechanism is not designed to procure IFA stock based on the demand of different population subsets, such as adolescents.

### 4.3 Study Limitations

The study was conducted in only eight districts of Pakistan. This could reflect sampling bias whereby inadvertent overrepresentation or underrepresentation of some geographies can take place. However, the findings from these districts are representative of the IFA landscape of the country since demand and supply‐side barriers reported are mostly the same across districts and lower levels of health service delivery. This is because the policy context for IFA service delivery is similar across all four provinces despite health being a devolved subject. The challenges and bottlenecks reported by districts across different provinces had underlying similarities which Maternal & Child Nutrition meant that the findings are applicable nationally. Moreover, given the linguistic and cultural diversity of Pakistan, it is probable that there were language and cultural biases. This is true when reaching out to vulnerable groups such as women, rural communities, and low‐income populations, as they may face unique challenges while accessing health care. However, to eliminate these biases, a diverse research team was formed who understood the linguistic, cultural and geographic sensitivities of the local populations to ensure that the needs and barriers experienced by everyone were understood and reported to ensure inclusive and equitable service delivery.

## 5 Conclusion

The iron‐folic acid bottleneck analysis in Pakistan has provided critical insights that hinder the effective implementation of nutrition programs and supplementation of iron‐folic acid. The analysis revealed a complex interplay of supply, demand, coverage, and enabling environment challenges.

On the supply side, the patchy availability of IFA supplements at the primary healthcare level and the affordability of supplements are identified as a significant bottleneck. Inconsistent supply exacerbated by bureaucratic processes and budgetary constraints hamper the continuous provision of supplements to the PLW. The supply is further impacted by low job satisfaction and excessive workload among healthcare providers. Demand‐side challenges, such as nutritional illiteracy among community members, cultural beliefs and taboos, and financial constraints, impact uptake and adherence to the supplements. Moreover, patriarchal values disempower women to make nutritional decisions, worsened by the lack of male engagement in women's health matters. On the governance level, the lack of prioritization of nutrition within health budgets and the lack of accountability and effective coordination among actors introduce system inefficacies, which hinder communication and awareness efforts.

Therefore, by addressing these critical bottlenecks and adopting the recommended strategies, the country can improve the delivery and uptake of iron‐folic acid supplements, ultimately leading to improvement in the maternal and child health outcomes. Effective collaboration between stakeholders, increased political commitment and evidence‐based interventions will be key to achieving success in this endeavor.

## Author Contributions

A.T., with the support of K.M. and S.S., conceived the design. M.I.K. and N.A. reviewed and provided input on the design. A.G. developed the tools. A.T. and A.G. conducted the data collection and analysis. S.R. wrote the first draft. B.A., W.A., S.R., A.K., A.A.B., and M.F. helped with subsequent drafts. All authors reviewed and contributed to the final version.

## Conflicts of Interest

A.T., M.I.K., and N.A. are directors at PHC Global. A.G., N.S., M.F., S.R., W.A., A.K.B., S.R., and B.A. are employees of PHC Global. K.M. is from the nutrition wing of Ministry of National Health Services, Regulations and Coordination (MoNHSRC). SS is from UNICEF Pakistan.

## Supporting information

Supporting information.

## Data Availability

Data that support the findings of this study are available from the corresponding author upon reasonable request.

## 1

Annex A: Guide for/Kii With District Program Managers Nutrition

Assalam‐o‐Alaikum, my name is (XYZ), and I want to discuss a very important study with you today. This is (other person's name) a colleague, and he/she will be taking notes on our discussion. Before we begin, I want to thank you for your agreement to participate in this study. We will discuss your knowledge regarding IFA supplementation, and the information you provide will remain confidential.

The objective of this study is to analyze bottlenecks to IFA supplementation and identify the main barriers to compliance with IFA supplementation among pregnant women. This analysis will provide the basis for informed nutrition policy, planning, and programming.

It is, therefore, important to understand your views on the implementation of the program, your perceived understanding of the needs of the program, and the bottlenecks to optimal implementation and the mitigation strategies to circumvent challenges.

Note: Do not read the underlined headings.

**Section: Awareness of the problem and its burden**

1. In your opinion, are there any nutrition problems among the population of this district? Are these problems different in genders? (Probe: female, males, children, emphasis on females if answered, why in females?)
2. In your opinion, is there malnutrition affecting pregnant and lactating women (PLW) in your district? If yes, is there a high burden? What are the factors which contribute to malnutrition in your district? (Probe: socio‐cultural, economic, programmatic others)
3. Do the nutritional needs differ between pregnant/lactating women, women of reproductive age (currently not pregnant), and adolescent girls? (probe: whether need is higher or lower in one of these groups)
4. What is the significance of nutrition during pregnancy/lactation and adolescence?
5. Generally speaking, is the target population aware of IDA? (Probe: do they know it's a disease? do they know it may adversely affect pregnancy outcomes? do they understand the need for good nutrition to circumvent adverse outcomes? Are they aware of specific remedies/interventions?).
6. How their knowledge regarding IFA supplementation could be improved?

**Section: Understanding of Program and Roles/Responsibilities**

1. What are the objectives of the Nutrition Program?
2. Could you please inform us about your general role and responsibilities? (probe: also specific to IFA supplementation)
3. How can you better work for IFA supplementation?
4. What is the role of the staff working for IFA supplementation at the district level? (probe: explain their role and responsibilities for IFA supplementation)
5. What are your views on the understanding level of staff supporting IFA supplementation in improving the health of women in general and PLW at the previously mentioned levels? (Probe: with which foods IFA should be taken with dosing and dosing time, compliance, etc.)
6. What could be done to improve their performance? (probe: better counseling skills, better/frequent training sessions, enhanced supportive supervision and monitoring, and increasing HR in the existing geographical area)
7. What are your views on the program implementation (probe: is the implementation robust? extent of geographical coverage? hard to reach groups such as nomads, ethnic groups, immigrants etc. covered?)
8. What is your suggestion for improving/strengthening this program? (Probe: expansion into uncovered areas, improving IFA availability, more resource allocation in this program, restructuring needed?)
9. Is there private sector engagement in the IFA supplementation program? (Probe: describe how it works. Could it be improved? If not engaged already, should it be engaged? And if already engaged, how can this be better integrated)
10. Do the private providers emphasize enough on IFA during pregnancy? If not, is there a mechanism to work with the providers (gynecologists/obstetricians) to emphasize IFA use during pregnancy?

**Section: Demand and Supply**

1. Please share your views on the consistency of IFA supply. (Probe: if there are stockouts, why do you think they happen? Do you also prescribe from stores outside when there's a stock out?)
2. How can these be prevented? (Probe: What is the backup plan for preventing? Does the plan need improvements or suggestions?)
3. How is the stock maintained? (Probe: Is the stock storage maintained under specific efficacy guidelines/measures? Who is responsible for keeping records? Does the storekeeper/dispenser record the stock out? Ask about the quality of staff, whether qualified or not, any pharmacist available or not)
4. Is there a logistic management system (LMIS) for record‐keeping of commodities? (Probe: Do you think it is an efficient way of addressing the supply barrier?)

**Section: Communication information**

1. Do you think there are any misconceptions, rumors, resistance to communication, or any other challenges to IFA uptake within the community? (Probe: How did you overcome these challenges and issues in raising awareness, educating, and explaining)
2. Was the program able to reach the population? What is the medium/approach to communicating information among the population in this program? (Probe: IEC material, mass media, social media, community mobilization/involvement)
3. What are/were the good things about the information communication component? What was not so good? How should this be improved?

Thanks for your participation. Your input and time are valuable additions to this study.

Annex B: Guide for Idi/kii With Provincial Program Managers Nutrition

Assalam‐o‐Alaikum, my name is (xyz) and I want to discuss a very important study with you today. This is (other person's name) a colleague and he/she will be taking notes on our discussion. Before we begin, I want to thank you for your agreement to participate in this study. We will be discussing about your knowledge regarding IFA supplementation, and the information you provide will remain confidential.

The objective of this study is to analyze bottlenecks to IFA supplementation and to identify the main barriers to access compliance to IFA supplementation within pregnant women. This analysis will provide the basis for informed nutrition policy, planning, and programming.

It is, therefore, important to understand your views on the implementation of the program, your perceived understanding of the needs of the program, and the bottlenecks to optimal implementation and the mitigation strategies to circumvent challenges.

Note: Do not read the underlined headings.

**Section: Awareness of the problem and its burden**

1. In your opinion, are there any nutrition problems among the population of this province? Are these problems different in genders? (probe: female, males, children, emphasis on females if answered)
2. In your opinion, is there malnutrition affecting pregnant and lactating women (PLW) in your province? If yes, is there a high burden? What are the factors which contribute to malnutrition in your province? (Probe: socio‐cultural, economic, programmatic others)
3. Do the nutritional needs differ between pregnant/lactating women, women of reproductive age (currently not pregnant), and adolescent girls? (probe: whether need is higher or lower in one of these groups)
4. What is the significance of nutrition during pregnancy/lactation and adolescence?
5. Generally speaking, is the target population aware of Iron Deficiency Anemia? (Probe: do they know it's a disease? do they know it may adversely affect pregnancy outcomes? do they understand the need for good nutrition to circumvent adverse outcomes? Are they aware of specific remedies/interventions?
6. How their knowledge regarding IFA supplementation could be improved?

**Section: Understanding of Program and Roles/Responsibilities**

1. What are the objectives of the Provincial Nutrition Program?
2. Could you please inform us about your general role and responsibilities? (probe: also specific to IFA supplementation)
3. How can you better work for IFA supplementation?
4. What is the role of the staff working for IFA supplementation at the Provincial Level? (probe: explain their role and responsibilities for IFA supplementation)
5. What are your views on the understanding level of staff supporting IFA supplementation in improving the health of women in general and PLW at the previously mentioned levels? (Probe: with which foods IFA should be taken, such as dosing and dosing time, compliance, etc.)
6. What could be done to improve their performance? (probe: better counseling skills, better/frequent training sessions, enhanced supportive supervision and monitoring, increasing HR in the existing geographical area)
7. What are your views on the program implementation (probe: is the implementation robust? extent of geographical coverage? hard to reach groups such as nomads, ethnic groups, immigrants etc. covered?)
8. What is your suggestion for improving/strengthening this program? (Probe: expansion into uncovered areas, improving IFA availability, resource allocation in this program? restructuring needed?)
9. Is there private sector engagement in the IFA supplementation program? (Probe: describe how it works. Could it be improved? If not engaged already, should it be engaged? And if already engaged, how can this be better integrated)
10. Do the private providers emphasize enough on IFA during pregnancy? If not, is there a mechanism to work with the providers (gynecologists/obstetricians) to emphasize IFA use during pregnancy?

**Section: Demand and Supply**

1. Please share your views on the consistency of IFA supply. (Probe: if there are stockouts, why do you think they happen? Do you also prescribe from stores outside when there is a stock out?)
2. How can these be prevented? (Probe: What is the backup plan for preventing? Does the plan need improvements and suggestions?)
3. How is the stock maintained? (Probe: Is the stock storage maintained under specific efficacy guidelines/measures? Who is responsible for keeping records? Does the storekeeper/dispenser record the stock out? Ask about the quality of staff, whether qualified or not, any pharmacist available or not)
4. Is there a logistic management system (LMIS) for the record‐keeping of commodities? (Probe: Do you think it is an efficient way of addressing the supply barrier?

**Section: Communication information**

1. Do you think there are any misconceptions, rumors, resistance to communication, or any other challenges to IFA uptake within the community? (Probe: How did you overcome these challenges and issues in raising awareness, educating, and explaining)
2. What is the medium/approach to communicating information among the population in this program? (Probe: IEC material, mass media, social media, community mobilization/involvement)
3. What are/were the good things about the information communication component? What was not so good? How should this be improved?

Thanks for your participation, and your input and time are valuable additions to this study.

Annex C: Guide for Idi/kii With Health Facility Medical Officer

Assalam o Alaikum, my name is (xyz), and I want to discuss a very important study with you today. This is (other person's name) a colleague, and he/she will be taking notes on our discussion. Before we begin, I want to thank you for your agreement to participate in this study. We will be discussing about your knowledge regarding IFA supplementation, and the information you provide will remain confidential.

The objectives of this study are to analyze bottlenecks to IFA supplementation and to identify the main barriers to access compliance to IFA supplementation within pregnant women. This analysis will provide ground for informed nutrition policy, planning and programming.

It is therefore important to understand your views on the implementation of the program, your perceived understanding of the needs of the program, the bottlenecks to optimal implementation, and the mitigation strategies to circumvent challenges.

Note to interviewer: Do not read the underlined text in headings.

**Section: Awareness of the problem and its burden**

1. In your opinion, are there any nutrition problems among the population of this area? Are these problems different in genders? (probe: female, males, children, emphasis on females if answered)
2. In your opinion, is there malnutrition affecting pregnant and lactating woman (PLW) in your area? If yes, is there a high burden? What are the factors which contribute to malnutrition in your area? (Probe: socio‐cultural, economic, programmatic, others)
3. Do the nutritional needs differ between pregnant/lactating women and women of reproductive age (unmarried and married with pregnancy)? (Probe: whether the need is higher or lower in PLW)
4. What is the significance of nutrition during pregnancy?
5. Generally speaking, is the target population aware of Iron Deficiency Anemia? (Probe: do they know it's a disease? do they know it may adversely affect pregnancy outcomes? Are there aware of any specific remedies/interventions?)
6. Do you believe that the target population understands the need for good nutrition or specifically IFA supplementation during pregnancy and women in general? (Probe: why do they think it is useful? How could their knowledge be improved? Could males be involved?)

**Section: Understanding of Program and Roles/Responsibilities**

1. Do you know about any program for nutrition supplementation run by govt in your area? (Probe: If yes, please elaborate. What is the program about? When was it started, and who is the target population? Responsible department and/or personnel for implementation of this program?)
2. Could you please inform us about your general role and responsibilities? (Probe: also specific to IFA supplementation, do you prescribe the supplements? who is accountable for dose monitoring and compliance)
3. How can you better work for IFA supplementation? (Probe: frequent training sessions, supportive supervision and monitoring, and improving commodity availability)
4. What is the role of the staff working with you at this facility for IFA supplementation? (Probe: explain their role and responsibilities for IFA supplementation)
5. What are your views on the understanding level of staff supporting IFA supplementation and nutrition support in improving the health of women in general and PLW at this facility? (Probe: dosing and dosing time, compliance, etc.)
6. What could be done to improve their performance? (Probe: frequent training sessions, supportive supervision and monitoring, increasing HR)
7. What are your views on the program implementation (probe: is the implementation robust? Are hard‐to‐reach groups such as nomads, ethnic groups, immigrants, etc. covered?)
8. What is your suggestion for improving/strengthening this program? (Probe: expansion into uncovered areas (increase coverage), improving IFA availability, resource allocation in this program? restructuring needed?)
9. Is there private sector engagement in the IFA supplementation program? (Probe: describe how it works. Could it be improved? If not engaged already, should it be engaged? And if already engaged, how can this be better integrated)
10. Do the private providers emphasize enough on IFA during pregnancy? If not, is there a mechanism to work with the providers (gynecologists/obstetricians) to emphasize IFA use during pregnancy?

**Section: Demand and Supply**

1. Please share your views on the consistency of IFA supply. (Probe: if there are stockouts, why do you think they happen? Do you also prescribe from stores outside when there's a stock out?)

2. How can these be prevented? (Probe: What is the backup plan for preventing? Does the plan need improvements or suggestions?)
3. How is the stock maintained? (Probe: Is the stock storage maintained under specific efficacy guidelines/measures? Who is responsible for keeping records? Does the storekeeper/dispenser record the stock out? Ask about the quality of staff, whether qualified or not, any pharmacist available or not)
4. Is there a logistic management system (LMIS) for the record‐keeping of commodities? (Probe: Do you think it is an efficient way of addressing the supply barrier?

**Section: Communication information**

1. Do you think there are any misconceptions, rumors, resistance to communication, or any other challenges to IFA uptake within the community? (Probe: How did you overcome these challenges and issues in raising awareness, educating, and explaining)
2. Was the program able to reach the population? What is the medium/approach to communicating information among the population in this program? (Probe: IEC material, mass media, social media, community mobilization/involvement)
3. What are/were the good things about the information communication component? What was not so good? How should this be improved?

Thanks for your participation; your input and time are valuable to this study.

Annex D: Guide for Focus Group Discussion With Wra (Plw And Adolescent Girls)

Assalam o Alaikum, my name is (xyz), and I want to discuss a very important study with you today. This is (other person's name) a colleague, and he/she will be taking notes on our discussion. Before we begin, I want to thank you for your agreement to participate in this study. We will be discussing your knowledge regarding IFA supplementation, and the information you provide will remain confidential. It is therefore important to understand your views on the implementation of the program, your perceived understanding of the needs of the program and the bottlenecks to optimal implementation, and the mitigation strategies to circumvent challenges.

If you do not have any questions, may we start?

Note to interviewer: Do not read the underlined text in headings.

1. How would you rate your current health status? (Probe: do you feel healthy or do you feel weak and/or tired? please explain in your opinion what reasons/factors are responsible for your current health status)
2. Why does one have to take care of one's health more so during pregnancy?
3. What additional nutritional demands are there in pregnancy? What are those sources other than food through which these demands can be met? (Probe: Are you aware of a nutrition supplementation program during pregnancy, any injectables? if yes, please explain both)
4. Do you have any idea of diseases that may result from nutritional deficiency? If yes, please share.
5. Are you satisfied with your current nutrition intake? If no, what are those essentials which you lack to consume?
6. Have you heard about anemia? If yes,
  a. Please discuss what that means.
  b. What are the factors behind it and how can we overcome this problem?
7. Did you visit a healthcare provider during your current/last pregnancy? If yes, why?
8. If not, why not?
9. In your opinion, when should you visit a healthcare provider/doctor after you become aware that you are pregnant? During which term did you visit? (First trimester, second, third)
10. Do you or other pregnant women seek consultation visits during their pregnancy? If yes, what is offered in those consultation sessions? (Probe: do they educate what IFA is? its consumption, dosing, duration of use (compliance), and adverse effects. Did they provide IFA during visits? Do they refer you to any facility for medicines (IFA/SUPPLEMENTS), etc.)
11. Were you prescribed any medicine during your visit?
  a. (Show them the IFA pack and then ask) do you know what this is? If yes,
  b. Did you receive this? Where did you get this from, and when?
12. Were you supplied enough IFA during your pregnancy? What did you do when you could not get an IFA? (Probe: ask about whether they contacted the health worker or they visited stores/health facilities or doctor)
13. Does a healthcare worker visit your house? If yes,
  a. How often does she visit?
  b. Does she provide IFA and emphasize its information and importance?
  c. Did she regularly provide IFA during your last pregnancy?
14. Did you use IFA in your last pregnancy? If she stopped providing IFA, how long was it for, and what was the reason behind it?
15. Do you have any concerns regarding IFA? Please share these concerns (if any). (Probe: any concerns regarding adverse/side effects of IFA/supply, any misconceptions, taboos, or rumors that you may have about.)
16. What do you think, under what conditions IFA must be taken? (Probe: its role in women's health, focus on a state of women when taken, i.e., in pregnancy or puberty, etc.)
17. Does anyone in your family or community discourage the use of IFA? If yes, why?
18. Do you feel any change in health status after taking IFA? (Probe: know the benefits)
19. What issues are there regarding the supply of IFA in your community? (Probe: quality and efficacy, affordability, human resources)
20. Did you ever encounter refusal in case of not having enough money to buy IFA? If yes, in your opinion why did this happen?

Thank you so much for your participation, the information you provided is a valuable addition in our study.

Annex E: Guide for Focus Group Discussion With Lhw/cmws

Assalam‐o‐Alaikum, my name is (xyz) and I want to discuss a very important study with you today. This is (other person's name) a colleague, and he/she will be taking notes on our discussion. Before we begin, I want to thank you for your agreement to participate in this study. We will be discussing about your knowledge regarding IFA supplementation and, the information you provide will remain confidential.

The objectives of this study are to analyze bottlenecks to IFA supplementation and to identify the main barriers to access compliance to IFA supplementation within pregnant women. This analysis will provide ground for informed nutrition policy, planning and programming.

It is, therefore, important to understand your views on the implementation of the program, your perceived understanding of the needs of the program, the bottlenecks to optimal implementation, and the mitigation strategies to circumvent challenges.

If you do not have any questions, may we start?

Note to interviewer: Do not read the underlined text in headings.

1. In your opinion, are there any nutrition problems among the population of this area? Are these problems different in genders? (Probe: female, males, children, emphasis on females if answered)
2. In your opinion, is there malnutrition affecting pregnant and lactating women (PLW) in your district? If yes, is there a high burden? What are the factors which contribute to malnutrition in your district? (Probe: socio‐cultural, economic, programmatic others)
3. Do the nutritional needs differ between pregnant/lactating women, women of reproductive age (currently not pregnant), and adolescent girls? (Probe: whether the need is higher or lower in one of these groups)
4. What is the significance of nutrition during pregnancy/lactation and adolescence?
5. Generally speaking, is the target population aware about Iron Deficiency Anemia? (Probe: do they know it's a disease, do they know it may adversely affect pregnancy outcome, are they aware of specific remedies/interventions?
6. How their knowledge regarding IFA supplementation could be improved?
7. Do you know about any program for nutrition supplementation run by govt in your district? (Probe: If yes, please elaborate. What is the program about? When was it started, and who is the target population? Responsible department and/or personnel for implementation of this program?)
8. Could you please inform us about your general role and responsibilities, which are also specific to IFA? (Probe: Do you visit households in your catchment area? If yes, how frequently? educating the target population in the catchment area regarding the nutrition need in general and IFA specifically? Do you prescribe the supplements? Who is accountable for dose monitoring?)
9. Do you keep a record when you provide IFA in your catchment area? (Probe: If yes, how? do you have a plan for whom to visit for IFA each month? If not, what are the reasons?)
10. What kind of methodology do you adopt to educate the population? Which method works best, and what else could be done to strengthen your work? (Probe: lecture, interactive sessions, community engagement activities, incentives?)
11. Other than the ways discussed, what else can help you work better for IFA supplementation? (Probe: frequent training sessions, supportive supervision and monitoring, improving IFA availability, increasing HR in the existing geographical area)
12. What are your views on the program implementation (Probe: is the implementation robust? What is the extent of geographical coverage? Are hard‐to‐reach groups such as nomads, ethnic groups, immigrants, etc. covered?)
13. What is your suggestion for improving/strengthening this program? (Probe: expansion into uncovered areas, improving IFA availability, resource allocation in this program? restructuring needed?)
14. Is there a mechanism to work with the providers (gynecologists/obstetricians) to emphasize IFA use during pregnancy?
15. Please share your views on the consistency of IFA supply. (Probe: if there are stockouts, why do you think they happen? Do you also prescribe from stores outside?)
16. How can these be prevented? (Probe: What is the backup plan for preventing? Does the plan need improvements, suggest?)
17. Did you or your team come across any misconceptions, resistance to communication, or any other challenges to IFA uptake within the community? (Probe: How did you overcome these challenges and issues in raising awareness, educating, and explaining)

Thank you so much for your participation; the information you provided is a valuable addition to our study.

Annex F: Guide for Focus Group Discussion For Community Members

Assalam‐o‐Alaikum, my name is Dr. Ali Turab, and I want to discuss a very important study with you today. This is Dr. Ammad A. Baig, a colleague who will be taking notes on our discussion. Before we begin, I want to thank you for your agreement to participate in this study. We will be discussing your knowledge regarding IFA supplementation, and the information you provide will remain confidential. It is, therefore, important to understand your views on the implementation of the program, your perceived understanding of the needs of the program, the bottlenecks to optimal implementation, and the mitigation strategies to circumvent challenges.

If you do not have any questions, may we start?

Note to interviewer: Do not read the underlined text in headings.

1. In your opinion, what is a healthy diet? What specifically composes healthy nutrition? (Probe: ask about the intake of the material, e.g., meats, vegetables, pulses/proteins, fruits, etc.)
2. Are you satisfied with your nutrition intake? If no, what are those essentials which you lack to consume? (Probe: What factors contribute to the nonavailability of the above‐mentioned items, e.g., low buying power due to poverty, item not available in your area, the item is available but is of very low/bad quality, socio‐cultural trends, e.g., tendency to eat more meat, rather than fruits and vegetables.)
3. Is there any difference in nutrition needs between men and women? If yes, what is the difference in nutritional requirements between a man and woman (unmarried and married without pregnancy)?
4. In your opinion, what is the healthy state of a mother? How does breastfeeding affect a mother's health? Do mothers need any other supplementation during breastfeeding?
5. Have you heard about anemia? If yes,
  a. Please discuss what does that mean
  b. What are the factors behind it and how can we overcome this problem?
  c. How may anemia impact pregnancy, any ideas?
6. What do you think is the burden of anemia and malnutrition in the population here? Is it higher in males or females or boys and girls? (Probe: Why do you think there is a difference)
7. Are you aware of IFA? If yes, under what conditions must IFA be taken? (Probe: its role in women's health, focus toward a state of women whether in pregnancy, lactating, or during puberty, etc.)
  a. Do you support IFA use? Why, please explain why do you think it is important to a woman's health? If not, why not explain?
  b. Please share your concerns about IFA (if any). (Probe: any adverse/side effects of IFA consumption)
8. Is there any nutrition supplementation program run by the govt in your area? If yes, please describe.
9. Does a healthcare worker visit your house? If yes,
  a. How often does she visit?
  b. What does she do during the visit? (Probe: Does she give information on IFA, food, and nutrition, and disburse IFA supplements?)
  c. In your opinion, does she adequately supply IFA?
10. When you visit a health facility, what is the process of getting medicine from that facility? (Probe: Who prescribes, who dispenses? Does anyone visit for supplies? Do they prescribe from outside the hospital?)
11. Were you or was your wife (phrase the sentence according to the gender of community member) supplied enough IFA during your pregnancy, please discuss? If not, what are the reasons?
  a. What's the option available when IFA is inaccessible? (Probe regarding buying from private practitioners or pharmacies outside of the government facility. Can you easily afford it or not? What other problems do you face when you have to buy from outside?)
12. Is it easy for you to contact the health worker and ask for a supplement (IFA)? If yes, how? If not, why not?
  a. Did you ever buy from the market? (Probe: try to find concerns regarding quality and efficacy)
13. Have you ever encountered refusal because you did not have enough money to buy an IFA? If yes, where did you register these complaints?
14. Are you satisfied with the efforts of the government in terms of IFA supplementation and nutrition as a whole?

Thank you so much for your participation, the information you provided is a valuable addition in our study.

Annex G: Nvivo Codebook

**Table:**

Name	Description	Files	References
**Acceptability**	**Acceptability deals with the demand and supply‐side factors which influence the nutritional interventions functionality, perception and how effectively does it meet the needs of the PLW** 1 **population.**	**65**	**616**
*Alternative Strategies to Address Anemia*	The alternative strategies to address anemia codes are the practices that direct and indirect beneficiaries have adopted to address iron deficiency anemia. This is noted in the absence or the presence of IFA^2^ supplements.	26	52
*Awareness‐IFA & Nutritional Knowledge*	The awareness of IFA and the nutritional interventions codes the levels and quality (i.e., adequate or inadequate) of awareness among the stakeholders.	10	423
Adequate Awareness	Awareness levels are coded ‘adequate’ if there is sufficient awareness remarked by various stakeholders of IFA and nutritional knowledge.	39	168
Inadequate Awareness	Awareness levels are coded ‘inadequate’ if there is insufficient awareness remarked by various stakeholders of IFA and nutritional knowledge. The code is explored in depth to develop a deeper assessment of key bottlenecks.	15	26
Lack of knowledge of Nutritional Interventions	This code represents insufficient knowledge of nutritional interventions, as observed by the stakeholders.	18	28
Lack of knowledge of Nutritional Status & knowledge	This code represents insufficient knowledge of nutritional knowledge, such as the importance of a balanced diet, malnutrition status, and increased nutritional requirements during pregnancy.	27	72
Literacy levels	This code represents the levels of literacy and the respective impact on the awareness levels of PLW.	8	10
Religious factors	This code represents the religious factors that limit understanding and awareness of nutritional supplements and information.	2	3
Knowledge of roles and responsibilities among public providers and leaders	The knowledge of roles and responsibilities among high‐level stakeholders' codes the questions posed regarding the responsibilities of nutritional actors involved at different levels of care.	21	116
* Initial Utilization*	Initial Utilization deals with the factors that impact the initial consumption of iron‐folic acid as a user or as a non‐user influenced by other PLW's intervention utilization concerns.	5	29
Concerns of side effects	This code represents the specific side effects and the impact on the acceptability of the intervention services.	11	14
Concerns with color or taste	This code represents the concerns related to color, taste and appearance of the iron‐folic acid supplement and its impact on the acceptability of the intervention services.	2	2
Concerns with IFA dosage	This code represents the concerns related to the IFA dosage prescribed by the HCP^3^ to the PLW and those cited by the HCP themselves based on empirical evidence.	3	5
Concerns with medicine shelf life	This code represents the concerns related to the supplement's shelf life and its impact on the acceptability of services, as it may hinder uptake or adherence to the intervention.	2	2
*Social Acceptability*	Social acceptability deals with the factors which impact the acceptance of the service among the community. These are mostly environmental and external.	50	91
Good acceptability	This code represents the factors which lead to good or adequate social acceptability of the intervention, as expressed by the stakeholders either directly or indirectly.	25	58
Lack of trust in LHWs	This code represents the factors that lead to a lack of trust in the services provided by the LHWs^4^ to the community.	4	5
Low adherence to IFA	This code represents the factors which impact the social acceptability leading to low adherence of IFA among the population, i.e., PLW.	3	5
Misconceptions IFA uptake	This code represents the misconceptions that act as barriers to the initial uptake of iron‐folic acid among the vulnerable population.	12	15
**Affordability**	Affordability deals with the financial aspect of the nutritional services which impact the consumption or utilization of iron‐folic acid among the PLW population in the districts and regions.	**20**	**33**
**Availability**	Availability deals with the factors that influence the availability of iron‐folic acid and other nutritional services with various stakeholders.	**65**	**448**
* Anemia testing*	This code represents the available anemia testing services in districts and regions.	11	16
* Essential Commodities*	Essential commodities cover the factors that influence the supply‐side components for iron‐folic acid, such as procurement, forecasting, funds, and the supply chain systems.	11	11
* Availability of IFA*	This code represents good or adequate availability of iron folic acid in the districts or regions.	25	68
* Unavailability of IFA and supply chain bottlenecks*	This code represents the bottlenecks in the supply chain system that impact the unavailability of the commodity in the health system.	27	81
Existing supply protocols	This code represents the gaps in the existing supply protocols.	9	14
IFA procurement	This code represents the gaps in the procurement protocols for iron‐folic acid supplements at the district and provincial levels.	12	21
Increase in demand and supply	This code represents the gaps in demand and supply, implying an increase in the factors.	3	4
Lack of funds to sustain supply	This code represents the factors that lead to a lack of funds to sustain supply in the supply chain system.	13	26
Lack of supply forecasting	This code represents the barriers to effective forecasting protocols that impact the supply of iron‐folic acid based on the demand.	2	3
Reliance on Manual Supply System	This code represents the manual aspects of the supply chain system that impact the supply chain processes and introduce critical delays.	6	9
Providers dependent on external supply chains	This code represents the dependence on the external supply chains to compensate for the internal supply chain gaps.	6	8
Reliance on external procurement agencies	This code represents the dependence on external procurement agencies to compensate for the internal procurement delays.	10	15
Stockout Report	This code represents the frequency of stockout events in the supply chain.	10	20
*Food availability in local markets*	Food availability in local markets covers the availability of nutrient‐rich foods in the local markets for the direct beneficiaries.	12	17
*Human Resources*	Human resources deals with the availability of human resources to support nutritional activities, such as supplementation and counseling services.	65	210
* HCP Counseling on IFA*	This code represents the bottlenecks in the counseling services provided by the stakeholders and the levels of satisfaction.	24	74
* Insufficient HR; overburdened LHW and LHV* 5	This code represents the factors that adversely impact the levels of job satisfaction for the human resources, such as burnout, excessive workload and inadequate financial remuneration.	16	41
* Quality of HR* 6	This code represents the quality of human resources on three aspects—knowledge of supplement among districts officers, lack of frequent capacity‐building training, and lack of knowledge among LHWs.	28	61
Lack of knowledge of IFA among district officers	This code covers the levels of knowledge among the district officers and the related barriers.	13	38
Lack of frequent capacity‐building among HCPs	This codec covers the factors negatively impacting the frequent capacity building or refresher training among HCPs.^7^	9	15
Lack of knowledge of IFA among community healthcare workers	This code covers the levels of knowledge among the community healthcare workers and the related barriers.	4	6
* Lack of Supportive Supervision*	This code represents the bottlenecks in the supportive supervision available to the human resource to support in supplementation of IFA to the community.	10	14
* Implementation service delivery*	This code covers the facets that impact the implementation service delivery of IFA through the healthcare workers in the district and regions.	17	31
Private sector engagement in IFA supplementation	This code represents the private sector's engagement in IFA supplementation and the related bottlenecks.	10	23
* Physical Access to Services*	Physical access to services deals with the bottlenecks impacting the physical access to iron folic acid supplementation services among the stakeholders.	7	10
**Coverage of Services**	**Coverage of services deals with the factors influencing the equitable, adequate and appropriate coverage of IFA services among the population, including the type of residence (urban or rural), geographical terrain and the gender of the population.**	**55**	**94**
* Adequate Coverage*	This code represents the adequate coverage of IFA services among the key districts and regions.	11	13
* Inadequate Coverage*	This code represents the inadequate coverage of IFA services among the key districts and regions.	18	30
* Lack of Targeted IFA Communication*	This code represents the lack of targeted communication, which impacts the quality of IFA information dissemination among the target population.	2	5
* LHW Community Mobilization*	This code represents the factors that negatively influence the LHW community mobilization activities in the districts or regions.	16	33
**Enabling Environment**	**Enabling environment deals with the determinants of social norms, such as factors related to societal practices, policies, legislation, governance and budget.**	**65**	**578**
* Budgetary Constraints*	This code represents the factors that influence or enable the budget for nutritional programs and services.	8	18
* Constitutional Changes*	This code covers the constitutional changes in Pakistan which have led to key changes in the nutritional programs and services in the districts or regions.	15	24
18th amendment	This code specifically represents the changes in nutritional services due to the 18th Amendment.	4	6
PC‐1	This code specifically represents the changes in nutritional services due to the PC‐1^8^ functionality, such as the approvals and expiry.	4	6
*Existing Intervention and Practices*	This code covers the existing nutritional interventions and practices in the districts and regions.	51	141
Current Modes of Communication	This code represents the bottlenecks in the current mode of nutritional communication between the PLW and community members.	11	30
LHW Program	This code represents the bottlenecks specific to the implementation of the LHW program in the district and regions.	12	26
Nutrition Program	This code represents the bottlenecks exclusive to the regional and district nutritional programs.	23	53
*Existing Policies or Legislation*	The existing policies and legislations inform the implementation of nutritional programs and the supplementation of IFA supplements.	13	23
Existing information systems mechanism	This code represents the bottlenecks in the information management systems relating to iron‐folic acid.	2	3
Other policies and legislation	This code represents other policies and legislation that create bottlenecks in IFA supplementation.	1	1
*Governance Challenges*	This code covers the governance challenges influencing the IFA demand and supply bottlenecks.	43	53
The absence of focused maternal nutritional program	This code represents the focused challenges with the absence of maternal nutrition programs in the district and the regions.	5	5
Competing PHC challenges and limited administrative capacity	This code represents the challenges in IFA supplementation due to the competing primary healthcare responsibilities of the healthcare workers and other administrative challenges in reaching the hard‐to‐reach groups.	5	6
Inadequate Infrastructural support and disaster preparedness	This code represents the infrastructural bottlenecks, the lack of disaster preparedness, and its impact on iron‐ folic acid supplementation.	6	13
Lack of collaboration with the private sector	This code represents the lack of collaboration with the private sector to conduct nutritional activities and related disruptions in the supply chain.	8	9
Poor utilization of information management systems	This code represents the poor utilization of the information management systems in the district and regions.	10	13
Short‐term programs and limited funding	This code represents the short‐term nature of the nutritional programs and limited funding and its impact on iron‐folic acid supplementation.	4	5
Social Norms	Social Norms cover the social and cultural aspects that lead to bottlenecks in iron‐folic acid supplementation.	65	319
Family support regarding IFA consumption	This code represents the lack of family support and related bottlenecks.	9	14
Health‐seeking behavior‐ANC^9^ Visits and delays	This code represents the bottlenecks in iron‐folic acid supplementation due to the health‐seeking behavior of pregnant and lactating women.	11	41
Internalized social norms‐dietary habits, norms	This code represents the internalized social norms related to dietary habits and cultural food practices, such as food taboos.	19	39
Internalized social norms‐patriarchal society	This code represents the internalized social norms related to patriarchal norms, which favor men's health over pregnant and lactating women.	14	18
Lack of health education and importance of a balanced diet	This code represents the lack of health education in the community and the resultant lower importance of a balanced diet.	26	60
Poor family planning practices	This code represents the poor practice of family planning and the resultant bottlenecks in iron‐folic supplementation.	9	16
Poverty and other socioeconomic issues	This code represents the bottlenecks in accessing iron‐folic acid due to the socioeconomic conditions of the community.	38	88
Prioritizing male children	This code represents the social norms that create preference for male children and its health impacts on the female.	12	36
**Recommendations**	**Recommendation deals with the stakeholder suggestions and comments for the highlighted demand, supply, coverage and enabling environment bottlenecks.**	**65**	**451**
* Adequate and dedicated infrastructural support*	This code represents the recommendations specific to enhancing the infrastructural support for nutritional interventions.	15	28
* Adopt an interdisciplinary, gender equitable approach*	This code represents the recommendations pushing for a more gender‐equitable and interdisciplinary approach to IFA supplementation.	8	11
* Collaboration with local and private organizations*	This code represents the recommendations pushing for effective collaboration between the private and the public sector nutrition interventions.	15	26
* Community Mobilization Sessions*	This code represents the recommendations pushing for community mobilization sessions, including raising awareness among the community.	24	54
* Conduct Training of healthcare providers*	This code represents the training of healthcare providers' recommendations, including counseling sessions.	31	89
* Digitize health systems*	This code represents the recommendations advocating for the digitization of manual health information systems to improve system efficiency.	4	4
* Engaging key community stakeholders*	This code represents the recommendations suggesting a deeper engagement of community stakeholders, such as male members, religious scholars, etc.	9	12
* Ensure Availability of iron folic acid*	This code represents the recommendations suggesting enhanced availability of iron‐folic acid through improved mechanisms.	14	36
* Ensure supportive supervision*	This code represents the recommendations to improve or enhance supportive supervision mechanisms for healthcare providers.	17	29
* Ensure sustainable funds*	This code represents the recommendations to ensure sustainable funds for improving iron‐folic acid service delivery.	7	8
* Improve supply chain mechanisms*	This code represents the recommendations suggesting an improvement in the supply chain mechanisms, including forecasting, procurement, and warehousing.	13	21
* Incentivize lady health workers*	This code represents the recommendations suggesting incentivization of lady health workers with suggested mechanisms.	3	3
* Increase awareness campaigns*	This code represents the recommendations suggesting an increase in awareness sessions or campaigns to improve nutritional literacy and health behavior.	17	29
* Increase Human Resource*	This code represents the recommendations related to increasing human resources to support the effective implementation of iron‐folic acid supplements.	30	44
* Invest in innovative approaches and small‐scale interventions*	This code represents recommendations suggesting an investment in innovative approaches and small‐scale interventions to enhance iron folic acid service delivery.	4	4
* Mass Media Campaigns*	This code represents recommendations suggesting mass media campaigns using various modes of communication to increase outreach.	19	34
* Strengthen primary healthcare facilities*	This recommendation suggests strengthening the primary healthcare services to address critical bottlenecks in iron‐folic supplementation.	3	5

^1^ PLW: Pregnant and lactating women.

^2^ IFA: Iron and folic acid supplements.

^3^ HCP: Health care personnel.

^4^ LHWs: Lady health worker.

^5^ LHV: Lady health visitors.

^6^ HR: Human Resource.

^7^ HCPs: Health care personnels.

^8^ PC‐1: Pakistan Planning Commission Form 1.

^9^ ANC: Antenatal care.

Annex H: Themes and Quotations

**Table:**

**Themes**	**Quotations**	***Source***
**Availability of Essential Commodities**	*In hospitals and in BHU there is a medical technician who gives us medicines. There is a store. Dr tells us which medicine is available at BHU and which medicine we have to take from the market.*	FGD Fathers
	*Mostly, women tell us that there is a lot of difference between the tablets they take from the market and the tablets that we provide them. They say those tablets that they purchased from the market are less effective.*	KII _LHW
	*If folic acid is not present, then they do have iron. We provide what we have.*	KII_LMO_Multan_Punjab
**Deficient Supply Distribution, Procurement, and Forecasting Mechanisms**	*Every LHW is responsible for 50 to 100 houses. The government provides only 30 Folic acid tablets. How can she distribute it in all homes!*	FGD Fathers
	*My compounder has all registers. He informs us about the medicine he brought that ‘Dr. these medicines have arrived’ for 3 months, but it isn't enough…*	FGD_LMO
	*BHU also get the IFA and iron tablets in less quantity. If they will get 2 or 3 packets, how can they give it to all patients?*	Baluchistan FGD Fathers
	*Here doctor checks 3000 patients a month. There are some BHUs where 300 patients visit, and they have 300 medicines as well. The government should give the medicines according to the patient ratio and needs.*	FGD Fathers
	*Whatever we quote, it depends on the clientele. Last month, we had 200 ANC with frequent revisits. We gave three during PNC and then three more visits and then there are either BD doses or OD dose so because it fluctuates, sometimes we have it sometimes we run out we don't determine a fix amount to procure.*	(KII_ BHU LMO_Sanghar)
	*We were not able to procure so much IFA supplements that can cater to the large adolescence population.*	KII_Nutrition Program_Punjab
	*We haven't had any medicine for more than 6 or 7 months. They used to give injections and tablets, whenever we get the stock, it is for only 3 months*	KII_LHW_Multan
**Lack of Funds to Sustain Supply**	*At hospitals even we are unable to provide IFA with continuity as we receive it for 2 or 3 months only. Recently we did not receive any stock for the year 2020 and 2021. This year is about to end, and they should give us the medicine, but they didn't give us one rupee, even one strip of medicine this year.*	KII_District Coordinator_Baluchistan
	*We thought we would ensure provision in areas where LHWs are completely absent i.e. uncovered areas, as identified by our DHOs but we were not given funds.*	KII_LHW_ Baluchistan
**Reliance on Manual Supply System**	*This is the manual system. This system will run approximately for 2 years because we have large registers so, they will last for two years. We often face the issues in keeping records this way.*	KII_District Cordinator_Baluchistan
**Provider Dependence on External Supply and Procurement Channels**	*Iron tablets were given by UNICEF 3 years ago. Now they aren't available.*	FGD LHW AJK
	*In Gilgit and Baluchistan, the tablet is not available so the LHWs, doctors, and LHVs, prescribe them to take it from the market if it is available.*	KII_LMO_KP
	*There isn't any engagement with the private sector on the provision of IFA supplements. They are costly, people can't afford.*	KII_Head of Health Department_Baluchistan
	*Now, as far as your micronutrients are concerned or deficiency of iodine. They [WFP] provide salt and now in the flour, they mix it and give it. Similarly, they do iron fortification.*	KII_DHO_AJK
	*It is manual. There is a storekeeper of the LHW program. He maintains it. It is not digital. though the data is entered in the computer.*	KII_DHO_Peshawar
**Supply Stockouts**	*I am not getting IFA from LHW from the past one month, I think it's maybe because they [LHW] don't get enough supplies.*	FGD 1 PLW
	*The quantity is not enough. The national program provides us with supplements but for the last 4 to 5 months we don't have medicines. Before 5 months, the supply of medicines was excellent but for 6 to 7 months and in between there is no medicine.*	KII_District Coordinator_Baluchistan
	*Sometimes we didn't get it, we aren't getting medicines for 3 years.*	KII_Department of Health_Baluchistan
	*We conduct nutrition weeks as well twice a year, but they were not carried out in COVID‐19. The last was in March and April.*	KII_PD Nutrition Program_Punjab
	*Dr asked me to buy it from drug store and the baji (LHW) also provides. When LHW doesn't have tablets then I buy it if I can.*	FGD_PLW
**Availability of human resources**		
**Overburdened Human Resource**	*The major issue in Gilgit is HR. Any program which is introduced, though there is enough money, there is no HR. The medical super attendants, directors or DHOs are given an additional charge for daily operations.*	KII_Director Planning_GB
	*There is a total of 33 LHWs where population size is one lac. Amongst this not even 2% of the PLW population will be covered effectively.*	KII_District Coordinator_Baluchistan
	*W2: I cater to 45 families. W3: I cater to 275 families. W1: I cater to 70 families. W4: I cater to 59, W5: and I cater to 75 Families.*	FGD_LHW
**Lack of Motivation**	*They have not given us the salaries for 5 months.*	KII_LHV_AJK
	*We haven't received diaries for many years. We manage everything ourselves. We are spending 1000 Rs for the diaries and register.*	FGD_LHW_AJK
	*Now you ask the woman [LHW] to take this cup from here to there, they will say what we will get?*	KII_LHW Cordinator_Baluchistan
	*One of the workers died on her duty for 3*rd *polio vaccination. From Muzaffarabad to Raj Pindian, everybody was testifying to her intense work as a vaccinator and health educator in the community. I think we should lessen their burden.*	KII_Program Coordinator LHW_AJK
**Lack of frequent capacity‐building training**	*I think they received training 2 or 3 years ago, maybe four. Everything is getting advanced and how would they know if they didn't get any training.*	KII_District Coordinator National Program_Baluchistan
	*Yes, storekeeper and pharmacist are available there, they record it.*	KII_Head of Health Department_Baluchistan
	*These two points are new for us that drinking tea after this tablet will not let iron absorb and it gets wasted. So, you can only drink tea after 2 to 3 hours of taking medicine.*	(FGD_LHV)
**Inadequate Supportive Supervision Services**	*Proper monitoring and supervision is needed for better performance.*	KII_Director Nutrition_GB
	*We did not have the facility of ultrasound but now we have it. Those who get to know about it often visit us. When it was not available, the mother got their ultrasound done from Chiniot DHQ.*	(FGD_LHV_AJK)
	*We have less technical skills on how to develop a message and which message will be effective to what extent, we have this deficiency.*	(KII_Program Coordinator_AJK)
**Quality of HCP Counseling Services**	*Yes, it is required but the burden is more. We often give less time to the patients because of it*	KII_LMO_KP
	*In our provisional setup, we provide counseling for family planning and minor elements, such as nutrition deficiency, IDA or worm infestation.*	KII_DHO_GB
	*Mostly they don't know what IFA is. They will only know if the LHWs are educated. There are lady health workers who are too old like 50 years, and they don't have metric degrees even.*	FGD_Fathers
	*The problem is that it causes constipation. LHWs were unable to tell them that it should be taken with laxatives; it should not be eaten alone to treat constipation.*	KII_LMO_KP
**Accessibility of services**		
**Physical Access to Services**	*There is a need to improve the access to the marginalized communities who live in congested, mountainous valleys.*	KII_Director Planning_GB
	*They take medicines of 6 to 7 persons from RHC as it is free. This way they cooperate as they cannot visit hospitals due to huge distance.*	KII_LHW_Multan
	*There are few limitations as it's a tribal area, you cannot communicate with the women directly. You can only reach them though their husband, brother or son. Though LHW program is beneficial, you need to struggle and work.*	KII_Head of Health Department_Baluchistan
**Factors affecting food availability**	*Baaji (LHW) tells me to consume what milk and fruits when she doesn't have medicine. We eat whatever is available at home.*	FGD 1 PLW
	*The fruit is‌ ‌not‌ ‌available‌ ‌in‌ ‌some‌ ‌places.‌ ‌There‌ ‌is‌ ‌1‌ ‌hour‌ ‌or‌ ‌2‌ ‌but‌ ‌they‌ ‌don't‌ ‌go‌ ‌to‌ ‌buy‌ ‌fruits, even‌ ‌if‌ ‌they‌ ‌have‌ ‌apples‌ ‌in‌ ‌their‌ ‌homes.*	FGD 4_Fathers_Baluchistan
	*For fisheries, we have Gwadar. We don't have any (supply) mechanisms here. Somehow it (fish) is available at Quetta in rotten condition but in remaining areas like Zoab, Qila Saifullah, Turbat, Dera Buqti and all deprived areas, people don't know (nutritional value). They don't like eating fish. They consider it a rotten thing as they have seen it in rotten form every time.*	KII_LHW Cordinator_Baluchistan
**Lack of awareness among direct beneficiaries**	*They aren't aware of what a balanced diet for them even would be. They just eat according to their preference.*	KII_LMO _Sanghar
	*In the districts I have worked in here, people are inclined towards spiritual remedies, so when an adolescent girl has shortness of breath, they push spiritual remedies because they believe she is attacked by souls (paranormal force).*	KII_LHW_Coordinator_Baluchistan
	*In rural areas, 10%–20% educated people know about balanced diet. The uneducated do not know about it but educated girls know.*	KII_Director Nutrition_GB
**Misconceptions on IFA Uptake**	*Once there was a rumor that the LHWs provide IFA to support family planning, as they were working on family planning as well.*	KII_Head of Health Dept._Baluchistan
	*A lot of them don't take these tablets or give it to their children and stop contacting LHWs due to fear of miscarriage or infertility in their daughters or sons.*	FGD_PLW 6_AJK
**Concerns with Initial Use**		
**Concerns with Taste, Color, and appearance**	*They eat wings of chicken and prefer it over iron tablets. If we give them the tablets, they throw them.*	KII_LHW_Chiniot
	*They often ask us why there is a picture of a mother and a child on it; we tell them it is not for family planning.*	KII_LHW_Multan
	*People say its standards are not the same as it becomes black.*	KII_DHO_AJK
**Concerns with IFA dosage**	*(LMO 1) I think its dose is less; (LMO 2) there is an issue of dose and; (LMO 3) yes I think these will be some issue of dose.*	FGD_LMO_ AJK
**Concerns with stomach issues**	*The only complaints we usually receive from the community are that continuous intake of IFA disturbs the stomach and if you consume it for more than 10 days, it affects gastric motility as it causes constipation. Sometimes people report complaints about loose stools or black stools. There are frequent complaints.*	KII_PD Nutrition Program_Punjab
**Concerns with shelf life**	*Medicines‌ ‌aren't‌ ‌available. The‌ ‌few‌ ‌medicines‌ ‌they‌ ‌give‌ ‌are‌ ‌near‌ ‌to‌ ‌expiry.*	FGD 4_Baluchistan
	*We are using old medicines because of budget issues and unabundant supply with short expiry.*	KII_Head of Health Department_Baluchistan
**Low Adherence Among Users**	*We don't have medicines like folic acid. If we don't have it, we ask mothers to purchase it from the market and due to this they often feel bad*.	FGD_LHV_AJK
	*Our girls were fearing that community demand from them, and when we are empty‐handed, we just talk, and everyone gives advice. People get fed up with verbal things, they will say either give us the money or IFA tablet. We are losing out trust due to no availability.*	KII_Program Coordinator LHW_AJK
	*Iron folic acid tablets that were coming were free of cost. If you give anything for free, they take it from LHWs in a paper and then throw it later. This is one thing that is why its usage is less.*	KII_Director Nutrition_GB
	*People (MILs, husbands, and mothers) in the uncovered area need a lot of awareness.*	KII_LHV_AJK
	*They don't need to take supplements. These supplements are harmful for babies, as these medicines are hot in nature, which causes acne/pimples to baby. Instead, mother needs to take milk and fruit.*	FGD_Fathers_1
	*They don't use properly. The complete half course and throw the rest of the tablets. Because they do not have education.*	FGD_LHV
**Lack of Targeted IFA Communication**	What we do is that we generalize our messages, and we think that our message will address all the issues for all the population in the same way. *This is not the right way of doing things. We should know their food routines are, the type of food they consume and understand if there is a specific issue lying in the food habit in specific area. Then we design a targeted campaign to change their food habits.*	KII_PD Nutrition Program_Punjab
**Initial utilization‐affordability**	*medicines that we purchased are not effective. They request for one tablet and ask us if we have one tablet, we should give them and they will feel better after eating even one tablet. Those people who do not use to taking medicines from us complain that they take costly medicines from the market and that is not effective.*	KII_LHW_Multan
	*It cost RS. 50 almost and it contains 100 tablets. And there are people who can't afford even this.*	FGD_LHV_AJK
	*I can say malnutrition is high because there is no food security. There is insecurity and poverty. Because people there is no industry here, we just have government jobs.*	KII_Director Nutrition_GB
**Coverage of IFA (Quality of Services)**		
**Differential access to services**	*Because it is a policy of our national program… nomads usually come in a way…like when a season starts, they arrive at mountains. They spend 2–3 months and leave. Our LHW cannot go there, and no one is available there.*	KII_District Coordinator National Program_Baluchistan
**Unstable coverage of services**	*And if we have the access as well, because most of our community do not have the road access as well, so sometimes it is difficult to reach them.*	KII_DHO/DC_Multan_Punjab
	*Sometimes once in a month or more, sometimes you don't and even if they visit, they don't have medicine. My house is even in her catchment area, but she has not visited it for 20 years almost.*	FGD_Fathers
	*The areas which are not LHW covered have an awareness problem.*	KII_DHO_Sanghar
	*Where there LHW does not visit, the women there even cannot visit hospitals. they don't know about the diet. They have no education and knowledge about it.*	KII_FGD_LHW_AJK
	*It is a holistic nutrition week That is being carried out by the support of lady health workers, so we supplement IFA to Lady health workers. In our covered area and in that nutrition week, we try to assess the nearest urban slums so at least they can be reached twice a year.*	KII_PD Nutrition Program_Punjab
**Enabling Environment (Social Norms)**		
**Social Norms & Lack of Family Support**	*During an FGD, a woman there told us that they are not allowed to take it from the market or mother‐in‐law tells the husband that it is not important for us.*	KII_LMO_KP
	*I know about it, but my family members do not know about it. Mother‐in‐law don't take care.*	PLW_FGD 4
	*They do eat the right foods but not according to what they are required. They have a lot of it, such as goat and cow's milk, ghee, lassi, eggs, chicken, etc. But they don't respond to it as well, but they are always working too much, so they don't eat healthily [sic].*	FGD_LHV_Sanghar
	*Our mothers‐in‐law say that it(tablets) is of no use, and we also never used it during our pregnancies. they should be told that why you there the land add fertilizer to grow crops on it.*	PLW FGD 4
	*Often women visit me and start crying that her mother‐in‐law doesn't allow how to eat.*	KII_WMO_Chiniot_Punjab
**Health seeking behavior**	*In 3rd month (1). 2nd 3rd month (2) Sometimes we must visit in the first month due to fever and vomiting (2) We visit in the third or fourth month for ultrasound (1). I am 5 months pregnant, but I have no check‐up done and took no medicine or drip. (1)*	FGD 4 PLW
	*If we give them the medicines for one month and ask them to visit again, they don't.*	KII_WMO_Chiniot
	*The fertility rate here is more than 2.5 so due to this there is a gap [to follow the] recommended birth spacing. It is recommended that after bearing one child, females prepare her body for the next childbirth with the help of supplementation, as the stores of iron deplete in them. If you will not prepare her body for the next childbirth, no doubt she will continue being iron deficient. They give birth to 4 to 5 children and may eat fortified.*	KII_Director Planning_GB
**Internalized social norms**		
**Dietary habits and patterns**	*The structure of our society is such that the best food at our homes is given to the guests and later the left over from that is eaten by males and at the third number the food is eaten by children and the left over from that is eaten by women. This is a cultural barrier*.	KII_Director Planning_GB
	*Food taboos are common. MIL and elders say that you don't eat this thing as its energetic*	KII_LMO_KP
	*I think male should be given a good diet because they work and earn. Our women are fat enough by sitting at homes Most of the women do not weigh less than 100 kg; they are already obese. They can hardly walk.*	FGD 3 _ Fathers
**Patriarchal Norms**	Here females are financially dependent on males. Men should also be involved because they often think that females are telling lies. *Females feel that their husbands do not trust them; they often bring them here as well. They ask us to counsel them.*	KII_LMO_Multan_Punjab
**Prioritization of male children**	*Girls observe the kind of food served at their homes to their father and to their brothers. That he [males] should eat leg piece.*	FGD_LHW_GB
	*They celebrate when a boy is born. They don't value a woman who gives birth to a girl*	FGD_LHW_GB
**Policies and Legislation**		
**18**th **amendment and OC‐1**	There are many financial crunches after the 18th amendment where social sector development is least prioritized.	KII_Program Coordinator Nutrition_AJK
	*I am claiming it in one way that our hope is on this PC‐1 for the nutrition in Azad Kashmir for IFA… so no not a single penny is allocated for it.*	KII_Program Coordinator Nutrition_AJK
	*We have WMOs at RHCs, whom we don't have for the current PC‐1. We have community midwives but now they are also excluded from the program.*	KII_PD Nutrition Program_Punjab
	*When we were making PC‐1, we took on board 1200 females on contracts. We thought we would do that in those areas which will be identified by our DHOs, where LHW are completely absent i.e. uncovered areas, as you were discussing, we will do this for them. we were not given funds.*	KII_LHW Program Cordinator_Baluchistan
**Existing Nutrition Intervention**	*There is no such nutrition program in Ziarat, its neglected in Ziarat.*	KII_Head of Health Department_Baluchistan
	*The nutrition program which is running in AJK is donor driven with partners UNICEF, World food program and partially WHO, and it is only in 5 districts. There is total 10 districts, in the remaining 5 districts there is nothing named as nutrition program that exist there.*	KII_Program Cordinator Nutrition_AJK
	*We have children under [sic] 5 years, we conduct a MUAC, check their height and weight. If they are suffering from malnutrition we refer them to OTP. For mothers there isn't anything of that sort, so we just give them iron folic acid.*	KII_LMO_Sanghar_Sindh
**Inefficient use of various modes of communication**	*Media can reach males easily but it can hardly reach females. They don't even have cell phones.*	KII_Head of Health Department_Baluchistan
	*When a malnourished child is referred to by a doctor and she receives nutrition, that's how they come to know about it as the awareness spreads through word of mouth. No mass awareness program.*	KII_DHO_Sanghar
	*In this important massage should be advertised in local language. Inform in local language what happens with iron and folic acid deficiency.*	KII_Director Nutrition_GB
**Other policies and legislation impacting access**	*One thing is, we include in our register people who stay somewhere for at least six months, some people settle in a place only for five to ten days, or for a month, if we register them, it becomes difficult for us to follow them.*	KII_DHO_GB
	*It is hard to reach nomads since they are constantly moving. They are beggars and not at home most of the day so it's hard to educate them. We need specific teams to keep check on the nomadic communities in their area.*	KII_DHO_Sanghar
**Budget and expenditure**	*There isn't any such budget that fills the national program gap.*	KII_DHO_AJK
	*We often face problems due to such emergency issues like COVID‐19, or due to budgeting issues.*	KII_DHO/DC_Multan_Punjab
	*Most of the programs are for a limited period. The project we are running witnessed the first two months without finances and whenever the project duration ends, we wait for the renewal of a project. Whether the renewal happens or not, that year is tough.*	KII_DHO/DC Multan
	*Nutrition is an expensive business. Its cost was so high, and the government said that our development budget is equal to your nutritional budget and asked you to reduce it.*	KII_LHW Cordinator_Baluchistan
**Governance**		
**Inadequate infrastructural support and disaster preparedness**	*They used to give us [IFA] before Corona… before COVID‐19 they did have IFA*	FGD 5_PLW_GB
	*All nutrition sites we had were closed and remaining stabilization centers were converted to COVID isolation wards. This is strange that it is not taken as a priority by us, i.e. malnutrition is taken as something at the bottom and considered as the last thing. It's not serious.*	KII_LHW Cordinator_Baluchistan
	*(LHW 1) we don't have our health house. (LHW 2) none of us have her health house because there are no rooms, there are not more rooms available. (W3) We conduct health houses in someone's drawing room. (LHW 4) I have made mothers room as a health house.*	FGD_LHW_Baluchistan
**Lack of collaboration between public and private sector**	*I think, it is good (to be done) at the urban level, but sometimes this causes doubling. we have such experience at one or two places, if they are involved, our work gets effected.*	KII_DHO_GB
	*We have LMIS and VLMIS, but it is not specific to these iron Folic acid supplements. They have their own way of managing which is normally done manually.*	KII_PD Nutrition Program_Punjab
	*P1: The LMIS is computerized at the district level but there is a manual, and we enter there. P2: it's manual from the facility.*	KII_Program Cordinator LHW_AJK

Annex I: Key Informant Interview Consent Form

Project Information

**Table:**

Project Title:	Project Number:
ERC Ref No:	Sponsor:
Principal Investigator:	Organization:
Location:	
Other Investigators:	Organization:
Location:	

Introduction

Greetings. I am here today representing Precision Health Consultants (PHC) Global, to collect some information from you regarding a health issue of great importance, which is the use of IFA and issues which hinder accessibility and supply of IFA to improve the health status of women and minimize the burden of maternal anemia. Therefore, you are invited to participate in this interview as your inputs will play a significant role in this research.

Purpose of the Study

By conducting this interview, we hope to understand the bottlenecks in the IFA supplementation. We want to know in details your views on factors which cause sub optimal usage in pregnant women/WRA and affect the availability, utilization and access to IFA.

Study Procedures

If you agree to participate, you will be interviewed at a private location. This interview may take approximately 1 h of your time. If you decide to participate, you will be asked to sign this form and you will be given a copy of the form to keep.

Benefits of Participation

There are no direct benefits to you from participating in this interview. However, the information we gather may help to improve the IFA supplementation program in Pakistan and will provide ground for informed nutrition policy, planning and programming that ensures sufficient availability and access of IFA to pregnant women in communities.

Risks of Participation

There are no physical risks from your participation in this interview. However, you may be asked a question that makes you uncomfortable. If you agree to participate, you can always choose not to answer any question that makes you uncomfortable. You can also stop the interview at any time.

Financial consideration

There is no financial compensation for your participation in this research.

Voluntarism

Your participation is completely voluntary. If you choose not to participate, your employment and the services you receive will not be affected in any way.

Confidentiality

The information you tell us will remain confidential. The data obtained from you will be used for analysis purposes and will be accessible to senior investigators only. The data will be de‐identified before analysis, and summary data of all participants will be shared with stakeholders only.

Storage of data

The data will be stored in secure cabinets and computers with password/s and will only be accessible to the investigators.

Right to refuse or withdraw

It is important that you understand the following general principles that will apply to all participants in this assessment:

1. Participation is entirely voluntary.
2. You may withdraw from this study at any time without penalty or loss of benefits.

Please feel free to ask any questions or to seek clarification if you do not understand any information. If you require any additional information regarding this study, please contact:

ABC PHC Global

Phone number: xxxx‐xxxxxx

Do you agree to participate? (tick one)

□ Yes                              □ No

I acknowledge that this assessment has been fully explained to me in a language that I understand and had the opportunity to ask questions which have been answered to my satisfaction. I agree voluntarily to participate in this study and understand that I have the right to withdraw at any time without penalty.

Participant's signature or thumb print:_______ Date:______________ Study ID:___________ Name of witness:______________ Signature of witness:_______________ Date:_____________

(If the participant cannot read and write)

I have explained the research to the participant and answered his/her questions to the best of my ability. I confirm that consent has been given freely

Investigator's Name__________________ Investigator's signature____________

Thank you for your time.
