# Supplementary material for: A Bottleneck Analysis of Iron and Folic Acid Supplementation Program in Pakistan
Source: Matern Child Nutr. 2025 Feb 11;21(3):e13797. doi: 10.1111/mcn.13797 (PMC12150130; doi:10.1111/mcn.13797)
Supplement: Supplementary file 1 — Supporting information. [file MCN-21-e13797-s001.docx]

**Supplementary Appendix—Quotations**

***Availability of essential commodities***

*“In hospitals and in BHU there is a medical technician who gives us medicines. There is a store. Dr tells us which medicine is available at BHU and which medicine we have to take from the market." (FGD Fathers)*

*" Mostly women tell us that there is a lot of difference between the tablets they take from the market and the tablets that we provide them. They say those tablets that they purchased from the market are less effective." (KII_LHW)*

*" If folic acid is not present then they do have iron. We provide what we have" (KII_LMO_Multan_Punjab)*

***Deficient supply distribution, procurement and forecasting mechanisms***

*Every LHW is responsible for 50 to 100 houses. The government provides only 30 Folic acid tablets. How can she distribute it in all homes! (FGD_Fathers)*

*"My compounder has all registers. He informs us about the medicine he brought that ‘dr. these medicines have arrived’ for 3 months, but it isn’t enough…" (FGD_LMO)*

*"BHU‌ ‌also‌ ‌get‌ ‌the‌ ‌IFA‌ ‌and‌ ‌iron‌ ‌tablets‌ ‌in‌ ‌less‌ ‌quantity. ‌‌If‌ ‌they‌ ‌will‌ ‌get‌ ‌2‌ ‌or‌ ‌3‌ ‌packets., how‌ ‌can‌ ‌they‌ ‌give‌ ‌it‌ ‌to‌ ‌all‌ ‌patients?" (Baluchistan FGD Fathers)*

*"Here doctor checks 3000 patients a month. There are some BHUs where 300 patients visit, and they have 300 medicines as well. The government should give the medicines according to the patient ratio and needs." (FGD Father)*

*"Whatever we quote, it depends on the clientele. Last month, we had 200 ANC with frequent revisits. We gave three during PNC and then three more visits and then there are either BD doses or OD dose so because it fluctuates, sometimes we have it sometimes we run out we don't determine a fix amount to procure." (KII_ BHU LMO_Sanghar)*

*"We were not able to procure so much IFA supplements that can cater to the large adolescence population." (KII_Nutrition Program_Punjab)*

*"We haven't had any medicine for more than 6 or 7 months. They used to give injections and tablets, whenever we get the stock, it is for only 3 months" (KII_LHW_Multan)*

**Lack of funds to sustain supply**

*"At hospitals even we are unable to provide IFA with continuity as we receive it for 2 or 3 months only. Recently we did not receive any stock for the year 2020 and 2021. This year is about to end, and they should give us the medicine, but they didn’t give us one rupee, even one strip of medicine this year." (KII_District Coordinator_Baluchistan)*

*"We thought we would ensure provision in areas where LHWs are completely absent i.e. uncovered areas, as identified by our DHOs but we were not given funds." (KII_LHW_Baluchistan)*

**Reliance on manual supply system**

*"This is the manual system. This system will run approximately for 2 years because we have large registers so, they will last for two years. We often face the issues in keeping records this way." (KII_District Cordinator_Baluchistan)*

**Provider dependence on external supply and procurement channels**

*"Iron tablets were given by UNICEF 3 years ago. Now they aren’t available." (FGD LHW AJK)*

*"In Gilgit and Baluchistan, the tablet is not available so the LHWs, doctors and LHVs, prescribe them to take it from the market if it is available." (KII_LMO_KP)*

*"There isn’t any engagement with the private sector on the provision of IFA supplements. They are costly, people can't afford." (KII_Head of Health Department_Baluchistan)*

*"Now, as far as your micronutrients are concerned or deficiency of iodine. They [WFP] provide salt and now in the flour, they mix it and give it. Similarly, they do iron fortification." (KII_DHO_AJK)*

*"It is manual. There is a storekeeper of the LHW program. He maintains it. It is not digital. though the data is entered in the computer." (KII_DHO_Peshawar)*

**Supply stockouts**

*"I am not getting IFA from LHW from the past one month, i think it's maybe because they [LHW] don't get enough supplies." (FGD 1 PLW)*

*"The quantity is not enough. The national program provides us with supplements but for the last 4 to 5 months we don’t have medicines. Before 5 months, the supply of medicines was excellent but for 6 to 7 months and in between there is no medicine." (KII_District Coordinator_Baluchistan)*

*"Sometimes we didn’t get it, we aren’t getting medicines for 3 years." (KII_Department of Health_Baluchistan)*

*"We conduct nutrition weeks as well twice a year, but they were not carried out in COVID-19. The last was in March and April." (KII_PD Nutrition Program_Punjab)*

*"Dr asked me to buy it from drug store and the baji (LHW) also provides. When LHW doesn't have tablets then I buy it if i can." (FGD_PLW)*

**Availability of human resources**

**Overburdened human resource**

*"The major issue in Gilgit is HR. Any program which is introduced, though there is enough money, there is no HR. The medical super attendants, directors or DHOs are given an additional charge for daily operations. [Additionally] due to the absence of any vertical program the burden of work is felt at the gross root level by LHW" (KII_Director Planning_GB)*

*"There is a total of 33 LHWs where population size is one lac. Amongst this not even 2% of the PLW population will be covered effectively. (KII_District Coordinator_Baluchistan)*

*"W2: I cater to 45 families. W3: I cater to 275 families. W1: I cater to 70 families. W4: I cater to 59, W5: and I cater to 75 Families." (FGD_LHW)*

**Lack of motivation**

*"They have not given us the salaries for 5 months." (KII_LHV_AJK)*

*"We haven’t received diaries for many years. We manage everything ourselves. We are spending 1000 Rs for the diaries and register." (FGD_LHW_AJK)*

*"Now you ask the woman [LHW] to take this cup from here to there, they will say what we will get?" (KII_LHW Cordinator_Baluchistan)*

*“One of the workers died on her duty for 3^rd^ polio vaccination. From Muzaffarabad to Raj Pindian, everybody was testifying to her intense work as a vaccinator and health educator in the community. I think we should lessen their burden" (KII_Program Coordinator LHW_AJK)*

**Lack of frequent capacity building training.**

*" I think they received training 2 or 3 years ago, maybe four. Everything is getting advanced and how would they know if they didn’t get any training." (KII_District Coordinator National Program_Baluchistan)*

*"Yes, storekeeper and pharmacist are available there, they record it." (KII_Head of Health Department_Baluchistan)*

*"These two points are new for us that drinking tea after this tablet will not let iron absorb and it gets wasted. So, you can only drink tea after 2 to 3 hours of taking medicine." (FGD_LHV)*

**Inadequate supportive supervision services**

*"Proper monitoring and supervision is needed for better performance." (KII_Director Nutrition_GB)*

*"We did not have the facility of ultrasound but now we have it. Those who get to know about it often visit us. When it was not available, the mother got their ultrasound done from Chiniot DHQ." (FGD_LHV_AJK)*

*"We have less technical skills on how to develop a message and which message will be effective to what extent, we have this deficiency." (KII_Program Coordinator_AJK)*

**Quality of HCP Counseling services**

*"Yes, it is required but the burden is more. We often give less time to the patients because of it." (KII_LMO_KP)*

*"In our provisional setup, we provide counseling for family planning and minor elements, such as nutrition deficiency, IDA or worm infestation. We prioritize the topical session which has more disease prevalence in the area so that more time is spent on that." (KII_DHO_GB)*

*"Mostly they don't know what IFA is. They will only know if the LHWs are educated. There are lady health workers who are too old like 50 years, and they don't have metric degrees even." (FGD_Fathers)*

*“The problem is that it causes constipation. LHWs were unable to tell them that it should be taken with laxatives; it should not be eaten alone to treat constipation." (KII_LMO_KP)*

*"Those who don’t have money, we ask them to eat apple, corn or eat such thing." (FGD_LHW_Baluchistan)*

***Accessibility of services***

**Physical access of services**

*"There is a need to improve the access to the marginalized communities who live in congested, mountainous valleys." (KII_Director Planning_GB)*

*"They take medicines of 6 to 7 persons from RHC as it is free. This way they cooperate as they cannot visit hospitals due to huge distance. I use a boat to visit there. (KII_LHW_Multan)*

*"There are few limitations as it’s a tribal area, you cannot communicate with the women directly. You can only reach them though their husband, brother or son. Though LHW program is beneficial, you need to struggle and work." (KII_Head of Health Department_Baluchistan)*

**Factors affecting food availability**

*"Baaji (LHW) tells me to consume what milk and fruits when she doesn’t have medicine. We eat whatever is available at home." (FGD 1 PLW)*

*"The‌ ‌fruit‌ ‌is‌ ‌not‌ ‌available‌ ‌in‌ ‌some‌ ‌places.‌ ‌There‌ ‌is‌ ‌1‌ ‌hour‌ ‌or‌ ‌2‌ ‌but‌ ‌they‌ ‌don’t‌ ‌go‌ ‌to‌ ‌buy‌ ‌fruits, even‌ ‌if‌ ‌they‌ ‌have‌ ‌apples‌ ‌in‌ ‌their‌ ‌homes." (FGD 4_Fathers_Baluchistan)*

*"For fisheries, we have Gwadar. We don’t have any (supply) mechanisms here. Somehow it (fish) is available at Quetta in rotten condition but in remaining areas like Zoab, Qila Saifullah, Turbat, Dera Buqti and all deprived areas, people don’t know (nutritional value). They don’t like eating fish. They consider it a rotten thing as they have seen it in rotten form every time." (KII_LHW Cordinator_Baluchistan)*

**Lack of awareness among direct beneficiaries**

*"They aren't aware of what a balanced diet for them even would be. They just eat according to their preference." (KII_LMO_Sanghar)*

*In the districts I have worked in here, people are inclined towards spiritual remedies, so when an adolescent girl has shortness of breath, they push spiritual remedies because they believe she is attacked by souls (paranormal force)." (KII_LHW_Coordinator_Baluchistan)*

*"In rural areas, 10-20% educated people know about balanced diet. The uneducated do not know about it but educated girls know." (KII_Director Nutrition_GB)*

**Misconceptions on IFA uptake**

*"Once there was a rumor that the LHWs provide IFA to support family planning, as they were working on family planning as well." (KII_Head of Health Dept._Baluchistan)*

*"A lot of them don’t take these tablets or give it to their children and stop contacting LHWs due to fear of miscarriage or infertility in their daughters or sons." (FGD_PLW 6_AJK)*

**Initial Use Concerns**

**Concerns with taste, color and appearance**

*"L: They eat wings of chicken. and they prefer it over iron tablets. If we give them the tablets they throw it. They don't eat it." (KII_LHW_Chiniot)*

*" They often ask us why there is a picture of a mother and a child on it; we tell them it is not for family planning." (KII_LHW_Multan)*

*"People say its standards are not the same as it becomes black." (KII_DHO_AJK)*

**Concerns with IFA dosage**

*"(LMO 1) I think its dose is less; (LMO 2) there is an issue of dose and; (LMO 3) yes I think these will be some issue of dose." (FGD_LMO_ AJK)*

**Concerns with stomach issues**

*"The only complaints we usually receive from the community are that continuous intake of IFA disturbs the stomach and if you consume it for more than 10 days, it affects gastric motility as it causes constipation. Sometimes people report complaints about loose stools or black stools. There are frequent complaints." (KII_PD Nutrition Program_Punjab)*

**Concerns with shelf life**

*"Medicines‌ ‌aren’t‌ ‌available. The‌ ‌few‌ ‌medicines‌ ‌they‌ ‌give‌ ‌are‌ ‌near‌ ‌to‌ ‌expiry." (FGD 4_Baluchistan)*

*"We are using old medicines because of budget issues and unabundant supply with short expiry.”*

*(KII_Head of Health Department_Baluchistan)*

**Low adherence among users**

*"We don't have medicines like folic acid. If we don't have it, we ask mothers to purchase it from the market and due to this they often feel bad. If we have the medicines, then patients will also visit us frequently." (FGD_LHV_AJK)*

*" Our girls were fearing that community demand from them, and when we are empty-handed, we just talk, and everyone gives advice. People get fed up with verbal things, they will say either give us the money or IFA tablet. We are losing out trust due to no availability " (KII_Program Coordinator LHW_AJK)*

*"They don't use properly. The complete half course and throw the rest of the tablets. Because they do not have education." (FGD_LHV)*

*"They don't need to take supplements. These supplements are harmful for babies, as these medicines are hot in nature, which causes acne/pimples to baby. Instead, mother needs to take milk and fruit." (FGD_Fathers_1)*

*"People (MILs, husbands, and mothers) in the uncovered area need a lot of awareness." (KII_LHV_AJK)*

*"Iron folic acid tablets that were coming were free of cost. If you give anything for free, they take it from LHWs in a paper and then throw it later. This is one thing that is why its usage is less." (KII_Director Nutrition_GB)*

**Lack of targeted IFA communication**

*" What we do is that we generalize our messages, and we think that our message will address all the issues for all the population in the same way. This is not the right way of doing things. We should know their food routines are, the type of food they consume and understand if there is a specific issue lying in the food habit in specific area. Then we design a targeted campaign to change their food habits." (KII_PD Nutrition Program_Punjab)*

**Initial utilization-affordability**

*"They say that the medicines that we purchased are not effective. They request for one tablet and ask us if we have one tablet, we should give them and they will feel better after eating even one tablet., Those people who do not use to taking medicines from us complain that they take costly medicines from the market and that is not effective." (KII_LHW_Multan)*

*"It cost RS. 50 almost and it contains 100 tablets. And there are people who can't afford even this." (FGD_LHV_AJK)*

*"I can say malnutrition is high because there is no food security. There is insecurity and poverty. Because people there is no industry here, we just have government jobs." (KII_Director Nutrition_GB)*

***Coverage of IFA***

**Quality of services**

**Differential access to services**

*"Because it is a policy of our national program… nomads usually come in a way…like when a season starts, they arrive at mountains. They spend 2-3 months and leave. Our LHW cannot go there, and no one is available there." (KII_District Coordinator National Program_Baluchistan)*

*"And if we have the access as well, because most of our community do not have the road access as well, so sometimes it is difficult to reach them." (KII_DHO/DC_Multan_Punjab)*

*"Sometimes once in a month or more, sometimes you don't and even if they visit, they don't have medicine. My house is even in her catchment area, but she has not visited it for 20 years almost." (FGD_Fathers)*

**Unstable coverage of services**

*“The areas which are not LHW covered have an awareness problem." (KII_DHO_Sanghar)*

*"Where there LHW does not visit, the women there even cannot visit hospitals. they don’t know about the diet. They have no education and knowledge about it." (KII_FGD_LHW_AJK)*

*"It is a holistic nutrition week That is being carried out by the support of lady health workers, so we supplement IFA to Lady health workers. In our covered area and in that nutrition week, we try to assess the nearest urban slums so at least they can be reached twice a year." (KII_PD Nutrition Program_Punjab)*

***Enabling Environment***

**Social Norms**

**Lack of family support**

*"During an FGD, a woman there told us that they are not allowed to take it from the market or mother-in-law tells the husband that it is not important for us. They do not buy." (KII_LMO_KP)*

*"I know about it, but my family members do not know about it. Mother-in-law don't take care." (PLW_FGD 4)*

*"Often women visit me and start crying that her mother-in-law doesn’t allow how to eat. " (KII_WMO_Chiniot_Punjab)*

*"Our mothers-in-law say that it(tablets) is of no use, and we also never used it during our pregnancies. they should be told that why you there the land add fertilizer to grow crops on it." (PLW FGD 4)*

*"They do eat the right foods but not according to what they are required. They have a lot of it, such as goat and cow's milk, ghee, lassi, eggs, chicken, etc. But they don't respond to it as well, but they are always working too much, so they don't eat healthily [sic]." (FGD_LHV_Sanghar)*

**Health seeking behavior**

*"In 3rd month (1). 2nd 3rd month (2) Sometimes we must visit in the first month due to fever and vomiting (2) We visit in the third or fourth month for ultrasound (1). I am 5 months pregnant, but I have no check-up done and took no medicine or drip. (1)" (FGD 4 PLW)*

*"If we give them the medicines for one month and ask them to visit again, they don't." (KII_WMO_Chiniot)*

*"The fertility rate here is more than 2.5 so due to this there is a gap [to follow the] recommended birth spacing. It is recommended that after bearing one child, females prepare her body for the next childbirth with the help of supplementation, as the stores of iron deplete in them. If you will not prepare her body for the next childbirth, no doubt she will continue being iron deficient. They give birth to 4 to 5 children and may eat fortified." (KII_Director Planning_GB)*

***Internalized social norms***

**Dietary habits and patterns**

*"The structure of our society is such that the best food at our homes is given to the guests and later the left over from that is eaten by males and at the third number the food is eaten by children and the left over from that is eaten by women. This is a cultural barrier." (KII_Director Planning_GB)*

*"Food taboos are common. MIL and elders say that you don’t eat this thing as its energetic" (KII_LMO_KP)*

*"I think male should be given a good diet because they work and earn. Our women are fat enough by sitting at homes Most of the women do not weigh less than 100 kg; they are already obese. They can hardly walk. " (FGD 3_Fathers)*

**Patriarchal Norms**

*"Here females are financially dependent on males. Men should also be involved because they often think that females are telling lies. Females feel that their husbands do not trust them; they often bring them here as well. They ask us to counsel them." (KII_LMO_Multan_Punjab)*

**Prioritization of male children**

*"Girls observe the kind of food served at their homes to their father and to their brothers.That he [males] should eat leg piece " (FGD_LHW_GB)*

*"They celebrate when a boy is born. They don’t value a woman who gives birth to a girl" (FGD_LHW_GB)*

***Policies and legislation***

**18^th^ amendment and OC-1.**

*"There are many financial crunches after the 18th amendment where social sector development is least prioritized." (KII_Program Coordinator Nutrition_AJK)*

*"I am claiming it in one way that our hope is on this pc1 for the nutrition in Azad Kashmir for IFA… so no not a single penny is allocated for it." (KII_Program Coordinator Nutrition_AJK)*

*"When we were making PC1, we took on board 1200 females on contracts. We thought we would do that in those areas which will be identified by our DHOs, where LHW are completely absent i.e. uncovered areas, as you were discussing, we will do this for them. we were not given funds." (KII_LHW Program Cordinator_Baluchistan)*

*"We have WMOs at RHCs, whom we don't have for the current PC1. We have community midwives but now they are also excluded from the program," (KII_PD Nutrition Program_Punjab)*

**Existing nutrition intervention and practices**

*"There is no such nutrition program in Ziarat, its neglected in Ziarat." (KII_Head of Health Department_Baluchistan)*

*"The nutrition program which is running in AJK is donor driven with partners UNICEF, World food program and partially WHO, and it is only in 5 districts. There is total 10 districts, in the remaining 5 districts there is nothing named as nutrition program that exist there." (KII_Program Cordinator Nutrition_AJK)*

*"We have children under [sic] 5 years, we conduct a MUAC, check their height and weight. If they are suffering from malnutrition we refer them to OTP. For mothers there isn't anything of that sort, so we just give them iron folic acid." (KII_LMO_Sanghar_Sindh)*

**Inefficient use of various modes of communication**

*"Media can reach males easily but it can hardly reach females. They don’t even have cell phones." (KII_Head of Health Department_Baluchistan)*

*"In this important massage should be advertised in local language. Inform in local language what happens with iron and folic acid deficiency." (KII_Director Nutrition_GB)*

*"When a malnourished child is referred to by a doctor and she receives nutrition, that’s how they come to know about it as the awareness spreads through word of mouth. No mass awareness program." (KII_DHO_Sanghar)*

**Other policies and legislation impacting access**

*"One thing is, we include in our register people who stay somewhere for at least six months, some people settle in a place only for five to ten days, or for a month, if we register them, it becomes difficult for us to follow them." (KII_DHO_GB)*

*"It is hard to reach nomads since they are constantly moving. They are beggars and not at home most of the day so it’s hard to educate them. We need specific teams to keep check on the nomadic communities in their area." (KII_DHO_Sanghar)*

***Budget and expenditure***

*"There isn’t any such budget that fills the national program gap." (KII_DHO_AJK)*

*"We often face problems due to such emergency issues like COVID-19, or due to budgeting issues." (KII_DHO/ DC_Multan_Punjab)*

*"Nutrition is an expensive business. Its cost was so high, and the government said that our development budget is equal to your nutritional budget and asked you to reduce it. " (KII_LHW Cordinator_Baluchistan)*

*"Most of the programs are for a limited period. The project we are running witnessed the first two months without finances and whenever the project duration ends, we wait for the renewal of a project. Whether the renewal happens or not, that year is tough." (KII_DHO/ DC Multan)*

***Governance***

**Inadequate infrastructural support and disaster preparedness**

*"They used to give us [IFA] before Corona… before COVID-19 they did have IFA" (FGD 5_PLW_GB)*

*"All nutrition sites we had were closed and remaining stabilization centers were converted to COVID isolation wards. This is strange that it is not taken as a priority by us, i.e. malnutrition is taken as something at the bottom and considered as the last thing. It’s not serious." (KII_LHW Cordinator_Baluchistan)*

*"(LHW 1) we don’t have our health house. (LHW 2) none of us have her health house because there are no rooms, there are not more rooms available. (W3) We conduct health houses in someone’s drawing room. (LHW 4) I have made mothers room as a health house." FGD_LHW_Baluchistan)*

**Lack of collaboration between public and private sector**

*" I think, it is good (to be done) at the urban level, but sometimes this causes doubling. we have such experience at one or two places, if they are involved, our work gets effected." (KII_DHO_GB)*

**Inefficient use of LMIS**

*" We have LMIS and VLMIS, but it is not specific to these iron Folic acid supplements. They have their own way of managing which is normally done manually." (KII_PD Nutrition Program_Punjab)*

*"P1: The LMIS is computerized at the district level but there is a manual, and we enter there. P2: it’s manual from the facility." (KII_Program Cordinator LHW_AJK)*
